# Different Mechanisms in Doxorubicin-Induced Neurotoxicity: Impact of BRCA Mutations

**DOI:** 10.3390/ijms26104736

**Published:** 2025-05-15

**Authors:** Kriti S. Bhatt, Aman Singh, Gursharan S. Marwaha, Naresh Ravendranathan, Inderbir S. Sandhu, Kristen Kim, Eesha Singh, Jefferson C. Frisbee, Krishna K. Singh

**Affiliations:** 1 Department of Medical Biophysics, Schulich School of Medicine and Dentistry, University of Western Ontario, London, ON N6A 5C1, Canada; kbhatt23@uwo.ca (K.S.B.); asing945@uwo.ca (A.S.); gmarwah4@uwo.ca (G.S.M.); nravendr@uwo.ca (N.R.); isandh@uwo.ca (I.S.S.); jkim2826@uwo.ca (K.K.); jfrisbee@uwo.ca (J.C.F.); 2 London Central Secondary School, London, ON N6B 2P8, Canada; 363178294@gotvdsb.ca; 3 Department of Anatomy and Cell Biology, Schulich School of Medicine and Dentistry, University of Western Ontario, London, ON N6A 5C1, Canada

**Keywords:** doxorubicin, BRCA1, BRCA2, cognitive impairment, neurodegeneration

## Abstract

The genotoxic drug doxorubicin (Dox) remains one of the most powerful chemotherapeutic options available for a wide range of cancers including breast, ovarian, and other cancers. However, emerging evidence links Dox treatment with chemotherapy-induced cognitive impairment, a condition that is popularly referred to as Dox-induced neurotoxicity or “chemobrain”, which limits the use of the drug. There are no specific treatments for Dox-induced neurotoxicity, only interventions to mitigate the neurotoxic effects of the drug. Accumulating evidence indicates that DNA damage, oxidative stress, dysregulation of autophagy and neurogenesis, inflammation, and apoptosis play central roles in Dox-induced neurotoxicity. Additionally, germline mutations in the tumour suppressor genes breast cancer susceptibility genes 1 and 2 (BRCA1 and BRCA2) increase the risk of breast, ovarian, and related cancers. BRCA1 and BRCA2 are distinct proteins that play crucial, unique roles in homologous recombination-mediated double-stranded break repair. Furthermore, BRCA1 and 2 mitigate oxidative stress in both neural cells and brain microvascular endothelial cells, which suggests that they have a critical role as regulators of pathways central to the development of Dox-induced neurotoxicity. Despite research on the effects of Dox on cognitive function, there is a gap in knowledge about the role of BRCA1 and BRCA2 in Dox-induced neurotoxicity. In this review, we discuss existing findings about the role of different mechanisms and the role of BRCA1 and BRCA2 in Dox-induced neurotoxicity, along with future perspectives.

## 1. Introduction

While cancer mortality is on the decline, the incidence rate for the top 10 cancers—breast, pancreas, prostate, and liver cancer to name a few—has been increasing [1,2]. Chemotherapy is used to kill cancer cells and enhance the effectiveness of other treatment modalities. One such potent chemotherapy drug is doxorubicin (Dox), an anthracycline antibiotic first isolated from randomly mutagenized *Streptomyces* (*S.*) *peucetius* species. Currently, Dox is commonly used to treat breast [3], ovarian [4], and bladder cancers [5,6], soft tissue sarcomas [7,8], leukemias [9], and other malignant tumours [10].

Dox prevents cancer progression mainly by inducing DNA damage and oxidative stress. The drug’s mechanism of DNA damage involves DNA intercalation, which leads to strand separation, DNA supercoils, and torsional stress, and topoisomerase II (Topo II) inhibition, which ensures continued torsional strain. Genomic stress inhibits DNA replication and transcription, ultimately causing cell death [11]. Dox induces oxidative stress through multiple intertwined pathways. Briefly, following administration, Dox is metabolized into doxorubicinol and subsequently converted into reactive intermediates including doxorubicin-semiquinone radicals, eventually generating reactive oxygen species (ROS) [12]. Enzymes such as nicotinamide adenine dinucleotide phosphate (NADPH) oxidases (NOXs), nitric oxide synthases (NOSs), and xanthine oxidase (XO) directly contribute to metabolizing Dox into the doxorubicin-semiquinone radical, further amplifying ROS production during Dox metabolism [12,13]. The combined effects of Dox-induced genomic and oxidative stress cause cancer cell death.

Dox is administered intravenously, and unfortunately, Dox’s cytotoxicity is not limited to malignant cells and can damage healthy cells as well, adversely affecting patient survival and quality of life. Cells activate DNA damage repair pathways such as error-free homologous recombination-mediated DNA damage repair (HDR) to repair Dox-induced DNA double-stranded breaks (DSBs). Breast cancer susceptibility genes 1 and 2 (BRCA1 and BRCA2) are two key tumor suppressor genes involved in this process [14]. Germline mutations in BRCA1 and BRCA2, common mutations in hereditary breast cancer, impair the ability of cells to effectively repair DSBs. BRCA1 and BRCA2 deficiency or HDR deficiency increases the susceptibility of healthy cells to Dox-induced damage accumulation and cytotoxicity [15,16,17,18]. Dox-induced cardiomyopathy (DIC) and myelosuppression are two of the most common dose-limiting toxicities that result from Dox chemotherapy [19,20].

Studies reveal that up to 70% of cancer patients receiving chemotherapy develop chemotherapy-induced cognitive impairment (CICI) or “chemobrain”, which negatively affects their quality of life [21]. Patients treated with Dox are not exempt from this fate and indeed suffer from Dox-induced neurotoxicity (DIN) and cognitive decline. In fact, Dox exposure significantly increases early and late neuronal cell death, making it one of the major drugs under investigation for cognitive decline [22]. Adjuvant endocrine therapy in breast cancer patients can contribute to furthering CICI because treatments such as aromatase inhibition prevent estrogen production, a hormone that is known to play a protective role in cognitive function [23]. Additionally, chemotherapy-induced neurotoxicity leads to a dose-limiting pathogenesis [24]. There are no specific treatments for DIN, only interventions to mitigate the neurotoxic effects of the drug, many of which will be discussed in this review. Moreover, there is currently a lack of biomarkers to identify patients at higher risk of DIN as they are still in the early stages of discovery.

DIN manifests through two primary mechanisms: directly and indirectly. The less common but direct mechanisms involve Dox either crossing the blood–brain barrier (BBB) to accumulate in the brain or reaching circumventricular organs in the brain such as the neurohypophysis, postremal area, median eminence, and subfornical organ which are characterized by a lack of BBB [25]. Previous work analyzing the accumulation of Dox in brain tissue reported between 0.1 and 0.3 ng of Dox per mg of fresh brain tissue with a partition coefficient (Kp) of less than 0.5 mL of Dox per gram of brain. The Kp represents the ratio of Dox’s concentration in brain tissue to blood, and a Kp value less than 1 indicates a low affinity of Dox to brain tissue, consistent with Dox’s limited ability to cross the BBB [26,27]. However, consistent localization of Dox has been observed in circumventricular organs [25]. The indirect mechanism of DIN is substantially more common and entails Dox-induced upregulation of tumor necrosis factor-alpha (TNF-α) in the systemic circulation. Elevated TNF-α can cross the BBB, leading to a 200% increase in brain TNF-α levels in some cases, and activate microglial cells, resulting in increased production of nitric oxide, mitochondrial dysfunction, and neuronal apoptosis [21,28,29].

**Figure 1:**
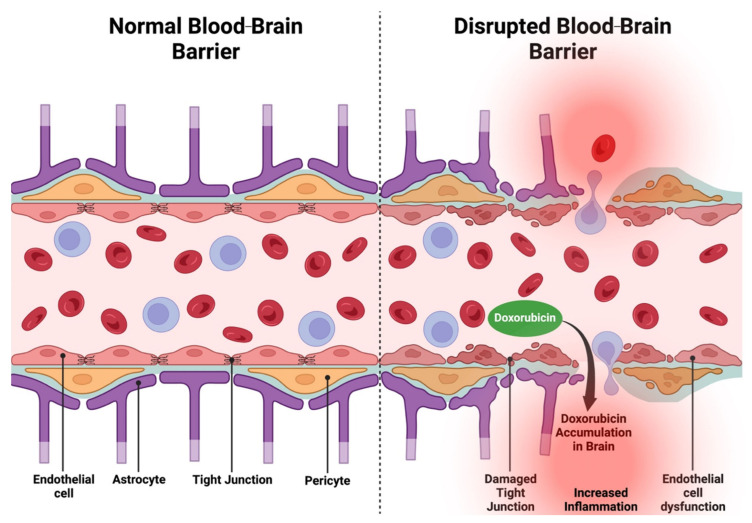
A comparison of a normal and disrupted blood–brain barrier. The normal blood brain barrier (**left**) contains tight junctions between endothelial cells and has supportive astrocytes and pericytes that maintain barrier integrity by regulating the transport of molecules and preventing harmful substances from entering the brain. Blood–brain barrier disruption (**right**), evident through damaged tight junctions and endothelial cell dysfunction, leads to increased permeability thereby permitting the accumulation of toxic substances such as Dox in the brain. Dox: Doxorubicin.

The BBB creates a controlled microenvironment for the central nervous system (CNS) by tightly controlling the passage of nutrients, oxygen, and waste products while preventing toxins, pathogens, and immune cells from infiltrating the CNS [30]. Brain microvascular endothelial cells (BMECs) that line cerebral capillaries to form the BBB express tight junctions, are non-fenestrated, do not have pinocytic activity, and have active transport mechanisms such as drug efflux [31]. These characteristics of the BBB limit the entry of undesirable substances like Dox into the CNS. Disruption of the BBB characterized by damaged tight junctions and endothelial cell dysfunction leads to increased permeability, potentially allowing toxic substances like Dox to accumulate in the brain (Figure 1) [32,33,34,35]. Recently, Dox was shown to cross the BBB via a newly discovered neural stem cell–blood vessel communication pathway where vessel-associated apical projections of neural stem cells of the dentate gyrus form direct membrane–membrane contacts with BMECs. These projections are greater than 30 nm in diameter and have ample vesicular activity, allowing substances to cross the intact BBB [36].

## 2. Breast Cancer Genes 1 and 2

As of 2020, breast cancer is the leading cause of global cancer incidence and the second leading cause of death from cancer in North American women [37]. Moreover, 5–10% of hereditary breast and ovarian cancer (HBOC) syndrome cases are usually due to mutations in BRCA1 and/or BRCA2 [38,39]. The BRCA genes encode two distinct, critical, and non-redundant mediators of HDR [14]. BRCA1 has roles in cell cycle checkpoint activation and DNA repair in HDR, while BRCA2’s key role is to mediate the core mechanism of HDR: recruit and activate effector proteins like RAD51 that catalyze strand invasion, exchange, and other key reactions of homologue recombination [14,40].

### 2.1. BRCA1/2 in DSB Repair

#### 2.1.1. Mechanisms of DSB Repair

DSBs are the most detrimental type of DNA damage. Cells need to conduct DDR to avoid genomic instability, particularly since genetic impairments are known to underlie premature aging, neurodegenerative diseases (cognitive impairment and developmental disorders), cancer, cardiovascular diseases, and autoimmune and metabolic disorders [41,42,43]. There are two DDR pathways for repairing DSBs: non-homologous end-joining (NHEJ) and HDR. Both mechanisms require ATM and ATR kinases to sense DNA damage and activate downstream players in the designated DDR pathway, including the histone H2AX and p53. Specifically, ATM and ATR phosphorylate the Ser15 residue of p53, which is a genotoxic phosphorylation site [44]. It should be noted that BRCC3 is a Lys63-specific deubiquitinating enzyme with cytoplasmic and nuclear roles. In the nucleus, BRCC3 forms the BRCA complex that recognizes the Lys63-ubiquitinated histone H2A and the phosphorylated H2AX at DNA lesion sites, ultimately facilitating recruitment of DNA repair proteins at DNA lesion sites for DDR [45].

NHEJ ligates broken fragments of DNA but is unable to recover genetic information lost to DSBs, which makes it an error-prone mechanism of DDR. NHEJ is also a more immediate response to DSBs that operates throughout the cell cycle, with the aim of rapidly resolving DSBs and preventing genomic instability that could lead to apoptosis [14,46]. NHEJ is initiated by 53BP1, recruited via ATM-mediated H2AX phosphorylation, which mediates the binding of the Ku70-Ku80 heterodimer (Ku) to the DSB lesion [47,48]. Upon Ku binding, DNA-PK stabilizes and aligns the broken DNA ends for ligation by the XRCC4-LIG4 complex.

In contrast, HDR is an error-free DDR process that is restricted to late S or G2 phases and requires a complete homologous template from a sister chromatid [14,46]. In HDR, the MRN complex conducts 5′ to 3′ DNA end resection to produce 3′ overhangs specific to the template and is required to activate ATM and ATR [14,49]. Much like how 53BP1 is the initiator for NHEJ, BRCA1 initiates HR upon activation by the MRN-ATM/ATR cascade. BRCA1 assists PALB2 in BRCA2-mediated loading of RAD51 onto the single-stranded 3′ overhangs, thereby initiating strand invasion to the correct regions on the homologous sister chromatid. Phosphorylation of the Ser988 site in BRCA1 by checkpoint kinases (CHKs) is required for the formation of this BRCA1-PALB2-BRCA2 complex [14].

**Figure 2:**
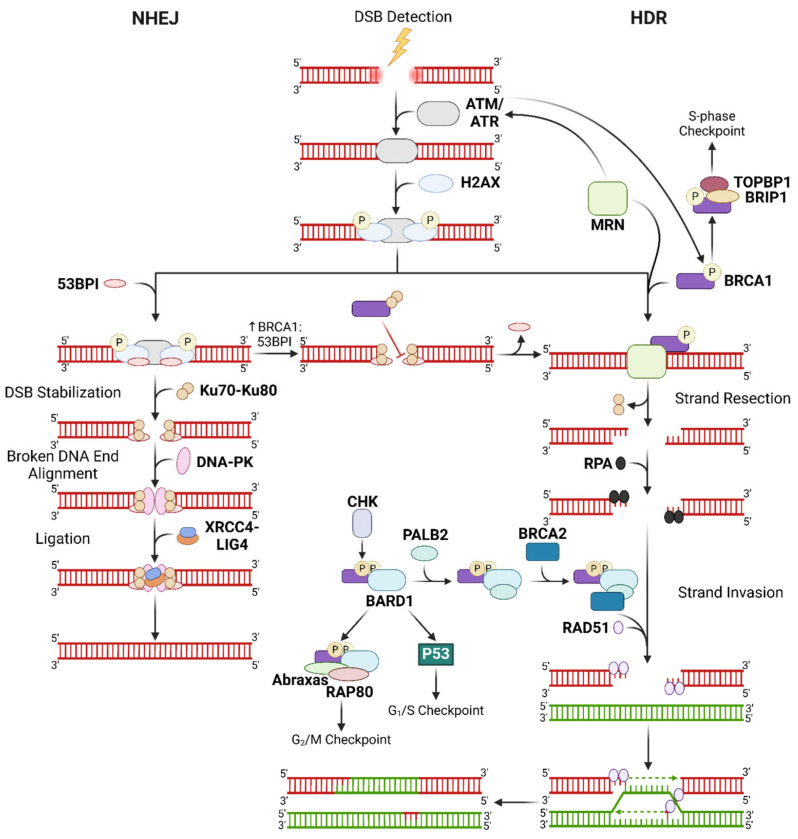
The roles of BRCA1 and BRCA2 in NHEJ and HDR. Both NHEJ and HDR begin with the detection of DSBs by ATM or ATR, which phosphorylate H2AX and enable it to bind to the broken DNA ends. To activate NHEJ, γH2AX recruits 53BPI which then recruits the Ku70-Ku80 heterodimer for DSB stabilization. Ku70-Ku80 then recruits DNA-PK to further stabilize the break and align the broken DNA ends. After alignment, the XRCC4-LIG4 complex ligates the broken ends. In HDR, the MRN complex plays a role in activating ATM or ATR and performing 5′ to 3′ DNA resection at the DSB when bound to BRCA1 that has been activated by ATM or ATR. HDR is preferred when there is greater bioavailability of BRCA1 relative to 53BPI, which leads to BRCA1 antagonizing 53BP1 and the replacement of Ku proteins at the broken DNA ends by the 3′ overhangs. RPA proteins then bind to the overhangs and are replaced by RAD51, which is recruited to the DSB via a BARD1-BRCA1-PALB2-BRCA2 complex. RAD51 then performs strand invasion, permitting repair of the DSB using a homologous DNA template. Phosphorylated BRCA1 can form a complex with BRIP1 and TOPBP1 to participate in S-phase checkpoints while BRCA1-BARD1 participates in activating the G_1_/S checkpoint via p53 phosphorylation and activating the G_2_/M checkpoint via the formation of a complex with Abraxas and RAP80. BRCA1: breast cancer susceptibility gene 1; BRCA2: breast cancer susceptibility gene 2; CHK: checkpoint kinase; HDR: homologous recombination-mediated DNA damage repair; HNEJ: non-homologous end-joining; TOPBP1: Topo II-binding protein 1.

Being the more sophisticated DSB repair mechanism, HDR is preferred over NHEJ when the cell has the necessary components. Accordingly, the HDR initiator BRCA1 is known to antagonize 53BP1, and BRCA1’s bioavailability relative to 53BP1 determines whether NHEJ or HDR is executed to repair DSBs [50]. DNA damage was found to facilitate BRCA1 recruitment to DSBs via interaction of its N-terminus with the Ku80 subunit, suggesting a more prominent role of BRCA1 in NHEJ that could explain its interaction with 53BP1 [14]. Moreover, the 3′ overhangs generated by the MRN complex replace Ku proteins with RPA which are then replaced by RAD51, inhibiting NHEJ [51]. Should DNA repair be unsuccessful, p53-mediated intrinsic apoptosis or cell cycle arrest is activated by DNA-PK and BRCA1 in NHEJ and HDR, respectively (Figure 2) [52,53].

#### 2.1.2. Role of BRCA1/2 in Modulating Oxidative Stress and Cellular Stress Reponses

Apart from participating in DDR, BRCA1 and 2 also modulate intracellular ROS levels by producing antioxidants. Specifically, BRCA1 directly interacts with and stabilizes NRF-2, which then upregulates gene expression of other antioxidants [54,55,56,57]. BRCA2 on the other hand indirectly upregulates antioxidant signalling via PALB2 stabilization and eventual NRF-2 activation [58]. Furthermore, BRCA1 plays a crucial role in the activation of cell cycle checkpoints where it complexes with several checkpoint factors. Activated ATM or ATR phosphorylates BRCA1 which enables the formation of the BRCA1-BARD1 complex that is involved in activating G_1_/S and G_2_/M checkpoints. At the G_1_/S checkpoint, the complex facilitates phosphorylation of Ser15 on p53 which induces the cyclin-dependent kinase inhibitor p21, ultimately activating the G_2_/M checkpoint. Effective G_2_/M checkpoint activation requires BRCA1 to form a complex with HR proteins RAP80 and abraxas in addition to BARD1. During the S-phase, BRCA1 forms the BRCA1-BRIP1-DNA Topo II-binding protein 1 (TOPBP1) megacomplex to interact with Topo II and regulate replication [14,59,60].

ATM also recruits scaffolding proteins such as XRCC1 which is involved in ROS-induced single-stranded DNA break repair [61]. This protein acts downstream of the enzyme poly-ADP ribose polymerase, which is a common target of breast cancer therapies due to the synthetic lethality of the treatment in the absence of BRCA1/2. On the other hand, fused in Sarcoma (FUS) is involved in transcription, DNA repair, and mediating shuttling between the nucleus and cytoplasm. Recently, the protein has also been found to play a role in DDR downstream of PARP [62].

## 3. Mechanisms of Direct DIN

**Figure 3:**
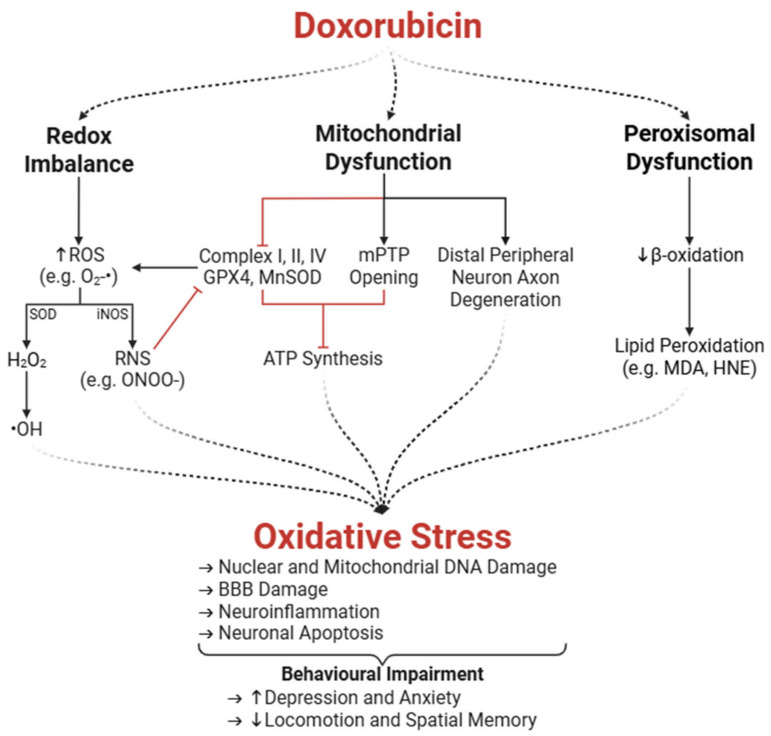
The mechanisms of Dox-induced neuronal oxidative stress. Dox initially leads to redox imbalance with excessive generation of ROS, including O_2_^−^•. O_2_^−^• are then broken into other ROS and RNS (H_2_O_2_, •OH, ONOO^−^), propagating the cycle of oxidative stress. Dox-induced ROS also cause mitochondrial dysfunction, which is characterized by the inhibition of the function of complexes I, II, and IV in the electron transport chain, reduced antioxidant enzyme activity (GPX4, MnSOD), and increased mPTP opening, all of which impair ATP synthesis. Dysfunctional electron transport chain complexes and antioxidant defenses exacerbate ROS production. On the other hand, Dox-induced mitochondrial dysfunction in peripheral neurons leads to distal neuron axon degeneration. Peroxisomal dysfunction also follows Dox treatment, where Dox leads to reduced β-oxidation and eventual lipid peroxidation, characterized by the byproducts MDA and HNE. Redox imbalance and mitochondrial and peroxisomal dysfunction culminate in oxidative stress, with a vast range of downstream cellular impacts and behavioural impairments. BBB: blood–brain barrier; GPX4: glutathione peroxidase 4; H_2_O_2_: hydrogen peroxide; HNE: 4-hydroxy-2-nonenal; iNOS: inducible nitric oxide synthase; MDA: malondialdehyde; MnSOD: manganese-containing superoxide dismutase; mPTP: mitochondrial permeability transition pore; O_2_^−^•: superoxide anions; ONOO^−^: peroxynitrite; RNS: reactive nitrogen species; ROS: reactive oxygen species; SOD: superoxide dismutase.

**Figure 4:**
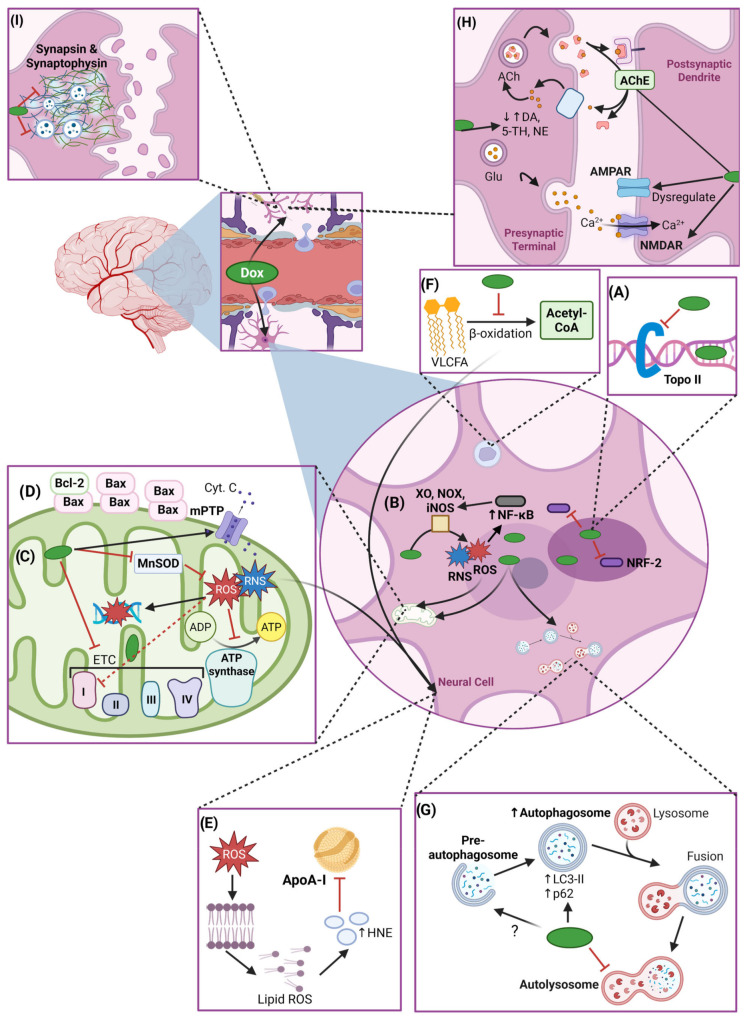
A schematic of the mechanisms of direct DIN. Dox-induced endothelial damage permits Dox to cross the BBB and exert its neurotoxic effects. (**A**) Dox induces DNA damage by intercalating with DNA and inhibiting Topo II. (**B**) Redox cycling of Dox by XO, NOX, and iNOS produces ROS which inflict oxidative stress in neurons. RNS are also formed by iNOS via Dox-mediated NF-κB activation. ROS and RNS lead to (**C**) mitochondrial dysfunction by inhibiting ATP synthesis and damaging mitochondrial DNA. Dox directly furthers mitochondrial dysfunction by inhibiting complexes in the ETC as well as mitochondrial antioxidant MnSOD, permitting ROS and RNS accumulation. (**D**) Dox-mediated accumulation of Bax and the opening of the mPTP permit Cyt. C release, triggering neuronal apoptosis. (**E**) Excessive ROS production in the mitochondria also leads to lipid peroxidation in membranes, and the byproduct of this process, HNE, impairs ApoA-I activity. Collectively, these effects impair neuronal membrane integrity. (**F**) Dox-induced cellular damage also includes peroxisomal dysfunction where the drug impairs β-oxidation of VLCFA, contributing to lipid peroxidation. (**G**) Dox-mediated impaired autophagy is evident through dysregulated pre-autophagosome initiation, increased autophagosome production via elevated LC3-II and p62 levels, and impaired autolysosome production (autophagosome turnover). (**H**) The drug also leads to neurotransmitter dysfunction through the dysregulation of NMDA and AMPA receptors and associated glutamate clearance and Ca^2+^ influx, unbalanced dopamine, serotonin, and norepinephrine levels, and impaired ACh turnover via dysregulated AChE activity. (**I**) NT dysfunction, through Dox-mediated reduction in the cytoskeletal proteins synaptophysin and synapsin, impairs synaptic plasticity and leads to synaptic dysplasia. 5-HT: serotonin; ACh: acetylcholine; AChE: acetylcholinesterase; AMPA: α-amino-3-hydroxyl-5-methyl-4-isoxazole-propionate receptor; ApoA-I: apolipoprotein A-I; BBB: blood–brain barrier; Ca^2+^: calcium; Cyt. C: cytochrome C; DA: dopamine; DIN: Dox-induced neurotoxicity; Dox: doxorubicin; ETC: electron transport chain; HNE: 4-hydroxy-2-nonenal; iNOS: inducible nitric oxide synthase; LC3-II: light chain 3 II; MnSOD: manganese-containing superoxide dismutase; mPTP: mitochondrial permeability transition pore; NE: norepinephrine; NF-κB: nuclear factor kappa beta; NMDA: N-methyl-d-aspartate; NO: nitric oxide; NOX: NADPH oxidase; ROS: reactive oxygen species; RNS: reactive nitrogen species; Topo II: Topoisomerase II; VLCFA: very-long-chain fatty acids; XO: xanthine oxidase.

Dox contributes to DIN through both direct and indirect mechanisms. The interconnection between these mechanisms is complex and requires comprehensive understanding to devise effective therapeutic interventions to prevent or treat DIN. This section focuses on Dox-induced direct neurotoxicity because despite having a limited ability to cross the BBB, the amount of Dox that does cross the BBB is sufficient to induce severe neurotoxicity, a process in which oxidative stress plays a key role (Figure 3). A schematic overview of mechanisms involved in direct DIN can be found in Figure 4.

### 3.1. Oxidative Stress

#### 3.1.1. Enzymatic Amplification of ROS and Redox Imbalance

Dox induces neurotoxicity through systemic and local oxidative stress mechanisms. As mentioned earlier, neural cells—neurons and glial cells—metabolize Dox, which eventually leads to the production of ROS, including superoxide anions (O_2_^−^•) [12,63]. In microglia, while ROS production is thought to be a part of their host defense function, it can also account for tissue destruction in the brain [64]. In fact, oxidative stress in all classes of macromolecules, such as proteins, nucleic acids, lipids, and carbohydrates, is found in neurons in diseased states [65,66]. O_2_^−^• are rapidly converted to hydrogen peroxide (H_2_O_2_), a less reactive compound, by superoxide dismutase (SOD). H_2_O_2_ can further form hydroxyl radicals (•OH), exacerbating oxidative stress [67]. Additionally, redox cycling of Dox between its quinone and semiquinone form and Dox-induced XO activity generates additional O_2_^−^• which serve as substrates for SOD [68]. Altogether, this creates a feedback loop that propagates oxidative damage due to disrupted cellular redox homeostasis [13,64].

Dox-derived ROS also upregulate inducible NOS (iNOS), a key transcription factor that regulates inflammatory and oxidative stress responses [69,70,71]. iNOS produces nitric oxide, which reacts with O_2_^−^• to form peroxynitrite (ONOO^−^), a highly reactive nitrogen species (RNS) also capable of damaging macromolecules [13,68]. This enzymatic pathway is particularly damaging in the brain’s vascular tissues and endothelium, where oxidative stress can impair the BBB and exacerbate neuroinflammatory responses, which is an indirect mechanism of DIN [64].

#### 3.1.2. Peroxisomal and Mitochondrial Dysfunction

As neurons have a high metabolic demand and limited regenerative capacity, they are especially vulnerable to oxidative damage, making the integrity of their antioxidant defense critical for maintaining brain health. Unfortunately, neuronal cells exhibit reduced expression of key antioxidant enzymes, such as catalase, glutathione peroxidase (GPX), SOD1, and manganese-containing SOD (MnSOD), implying poor antioxidant defenses compared to other tissues [72,73,74,75,76]. In particular, neuronal catalase levels are approximately thirty-times lower than those in hepatocytes, which limits the brain’s capacity to neutralize H_2_O_2_ and excessive ROS induced by Dox [77,78]. Additionally, Dox disrupts peroxisomal β-oxidation of very-long-chain fatty acids, leading to their accumulation. This not only contributes to lipid peroxidation, the process by which oxidants attack lipids to generate more free radicals, but also affects neuronal membrane integrity [79]. Indeed, increased levels of lipid peroxidation byproducts malondialdehyde (MDA) and protein-bound 4-hydroxy-2-nonenal (HNE) are observed in Dox-treated hippocampal tissues [80,81]. Combined, these effects of Dox chemotherapy compromise neuronal integrity and lead to neuronal apoptosis [64,82].

Beyond peroxisomal impairment, Dox also disrupts mitochondrial function, further exacerbating oxidative stress and energy deficits in neurons. Mitochondrial dysfunction is a hallmark of Dox-induced oxidative stress in the brain. Dox and ONOO^−^ inhibit complex I (a NOX enzyme) in the electron transport chain, which leads to excessive ROS generation during redox cycling at complex I, reducing the oxygen consumption rate and ultimately impairing ATP synthesis [69,83,84]. Mitochondrial ROS also damage mitochondrial DNA and compromise mitochondrial function and neuronal membrane integrity [69,85]. The oxidative insult is compounded by the high glucose turnover in the brain and the reduced capacity of neurons to counteract ROS [77,84]. Elevated oxidative stress correlates with reduced Bcl-2 and elevated Bax levels in hippocampal tissues, indicative of a pro-apoptotic shift [86].

Dox also induces the opening of the mitochondrial permeability transition pore (mPTP) which disrupts the mitochondrial membrane potential and ATP production, eventually leading to cell death via the release of cytochrome c [87]. Such mitochondrial dysfunction is also correlated with premature aging, thereby increasing the risk of neurodegenerative diseases [88]. Pharmacological inhibitors of mPTP prevent mitochondrial swelling, membrane potential dissipation, and the release of pro-apoptotic factors [85].

Given the mitochondria’s role as both a major source and target of ROS, specialized antioxidant enzymes like MnSOD are vital for neutralizing oxidative threats within these organelles. MnSOD is an analog of SOD located in the mitochondrial matrix that catalyzes the dismutation of O_2_^−^• into H_2_O_2_ and molecular oxygen [69]. Dox inhibits the activity of MnSOD and other mitochondrial enzymes (complex I, II, IV, and GPX4) through ONOO^−^-mediated nitration of their tyrosine residues [89]. This loss of MnSOD function contributes to an accumulation of O_2_^−^•, creating another feedback loop of oxidative damage [64]. Interestingly, iNOS knockout (iNOSKO) mice were protected from Dox-induced mitochondrial dysfunction by preserving MnSOD activity. Pharmacological inhibition of iNOS using N*ω*-Nitro-L-arginine methyl ester (L-NAME) further confirmed these findings [90]. Combined, the various oxidative stress-associated mitochondrial insults create a feedback loop that contributes to neuronal apoptosis, ultimately establishing oxidative stress as a central mediator of DIN.

#### 3.1.3. Mitochondrial Dysfunction in the Peripheral Nervous System

While much of the focus on DIN centers on the CNS, it is important to recognize that the peripheral nervous system (PNS) is also susceptible to oxidative damage and mitochondrial dysfunction. Dox-induced PNS toxicity is a significant concern due to its debilitating effects on sensory and motor functions. Dox-induced oxidative stress damages peripheral neurons, particularly sensory neurons in the dorsal root ganglia, eventually leading to axonal degeneration and neuronal apoptosis [91]. Additionally, Dox reduces antioxidant defenses in peripheral nerves [80], and oxidative damage in dorsal root ganglion neurons leads to axonal degeneration and impaired nerve conduction, resulting in sensory neuropathy [92,93]. The loss of mitochondrial integrity further disrupts energy-dependent axonal transport, leading to distal axon degeneration—a phenomenon characteristic of “dying-back neuropathy” observed in Dox-treated patients [92]. Moreover, Dox impairs calcium (Ca^2+^) homeostasis and alters NOS activity, both of which are critical for maintaining peripheral nerve function [94]. Alpha-lipoic acid (ALA) has been shown to mitigate these effects by reducing oxidative stress and preserving mitochondrial integrity in dorsal root ganglion neurons, offering potential neuroprotection against chemotherapy [93]. Collectively, these findings emphasize that Dox-induced peripheral neurotoxicity results from a complex interplay of oxidative stress, mitochondrial dysfunction, and impaired axonal transport.

#### 3.1.4. Neurobehavioural Impairments Associated with Oxidative Stress

Oxidative stress and the resulting neuronal damage not only impair cognitive and motor functions but also contribute to emotional and behavioural disturbances. Significant depression-like symptoms characterized by increased immobility in the forced swim test (FST) and reduced sucrose preference in the sucrose preference test (SPT) are observed in Dox-treated rats [72,95]. Other studies reported significant deficits in locomotive behaviour following Dox treatment [75]. The cerebellum is responsible for motor coordination; thus, oxidative stress and subsequent neuronal death in the cerebellum can help explain locomotive impairments detected in animal models and in clinical settings [75]. The elevated plus mase (EPM) test and open field test (OFT) revealed prominent anxiety-like behaviours in Dox-treated rats [72,95,96].

Neuroprotective interventions have demonstrated efficacy in ameliorating these deficits. Antioxidants and anti-inflammatory agents such as diphenyl diselenide (DPDS) and quercetin (QE) reversed motor coordination deficits and normalized anxiety- and depression-like behaviour in rats [75,95]. Coenzyme Q10 (CoQ10) is a ubiquitously expressed antioxidant and anti-inflammatory agent whose expression is reduced upon Dox exposure. CoQ10 supplementation improved neuronal function by attenuating Dox-induced inflammatory markers and increasing antioxidant pathways in neuronal cells due to CoQ10’s ability to cross the BBB. Moreover, the EPM and OFT revealed significantly reduced anxiety-like behaviours and partially restored locomotion with co-treatment of Dox-treated rats with CoQ10 [97]. dl-3-n-Butylphthalide (dl-NBP), a compound used for acute ischemic stroke treatment, reduced Dox-associated depressive behaviours in the FST and SPT [72]. Dox-treated rats displayed hindered performance in locating the hidden platform in the Morris Water Maze (MWM) test and spent less time in the target quadrant during probe trials, demonstrating negative consequences in spatial memory. However, rats co-treated with melatonin exhibited improved MWM performance, with reduced latency to the platform and increased time in the target area. These results were linked to Dox-induced oxidative stress which led to hippocampal damage and melatonin’s anti-oxidative and anti-inflammatory properties, mitigating damage [98].

#### 3.1.5. Therapeutic Interventions to Mitigate DIN-Associated Oxidative Stress

Given the central role of oxidative stress in the pathogenesis of DIN, a variety of therapeutic strategies have been investigated to either neutralize ROS or bolster antioxidant defenses. NOX inhibitors such as apocynin and diphenylene iodonium showed efficacy in reducing NOX-derived ROS production, while mitochondrial protectants like CoQ10 and mitoquinone supported ATP synthesis [13,97]. Antioxidant supplementation with ROS scavengers N-acetylcysteine (NAC), xanthone, and edaravone improved the adverse neural impacts of Dox by reducing the levels of iNOS, HNE, and other oxidized and nitrated proteins [79,99,100]. Similarly, antioxidant supplementation with omega-3 polyunsaturated fatty acids (ω-3 PUFAs) and berberine increased the activity of key antioxidant enzymes, reduced neuronal apoptosis, and preserved neural morphology and function in the cerebral cortex and hippocampus [70,101,102]. On the other hand, co-treatment with luteolin reduced XO enzyme activity, and pro-inflammatory and pro-apoptotic marker activity in the cerebrum, cerebellum, and hypothalamus of Dox-treated rats [74]. Osthole, a plant-based chemical compound, was reported to have neuroprotective effects against traumatic brain injury and Dox-induced apoptosis in human breast carcinoma cells [103,104]. Pretreatment of osthole to Dox-treated neuronal cells revealed increased neurogenesis and cell viability and reduced Dox-mediated ROS production and neuronal apoptosis [105]. Evidently, osthole is a promising neuroprotective agent and warrants in vivo investigation of its neuroprotective properties to elucidate its translational value.

### 3.2. Impaired Neurogenesis and Altered Neural Cell Morphology

In addition to inducing neurotoxic oxidative stress, Dox exposure impairs neurogenesis—the process by which new neurons are formed in the brain—further contributing to cognitive and behavioural deficits observed after chemotherapy. Adult neurogenesis and its role in learning and memory is emerging as a highly active area of research [106]. Focus is given to the hippocampal dentate gyrus and subventricular zone, two regions in the brain which continue to participate in neurogenesis well into adulthood, although Dox-induced damage to the cerebral cortex has also been reported [107].

#### 3.2.1. Dox-Mediated Impaired Neurogenesis

Dox impairs neurogenesis through multiple mechanisms, including reducing the proliferation and survival of neural progenitor cells and dysregulating key molecular pathways involved in neuron development. Studies in Dox-treated mice and rats revealed impaired neurogenesis through reduced expression of markers of immature neurons (doublecortin)—in the dentate gyrus—and proliferation (Ki67), mitochondrial dysfunction, and dysregulation of extracellular signal-regulated kinase and protein kinase B (also known as AKT) activity, all of which play important roles in neurogenesis signalling pathways [108,109,110]. Additionally, Dox treatment reduced the proliferation and survival of neural progenitor cells in the hippocampus. Due to the hippocampus’ critical role in mood regulation, impaired hippocampal neurogenesis is correlated with anxiety-like behaviours [111]. Dox-induced oxidative stress and inflammation also contribute to neural stem cell damage, further attenuating neurogenesis [109,112]. Interestingly, chronic nicotine treatment normalized cell proliferation in the hippocampus of Dox-exposed rats [113]. Ultimately, the reduction in hippocampal neurogenesis explains a significant component of CICI, highlighting the hippocampus as a vulnerable target of neurotoxicity. Understanding the mechanisms of neurogenesis suppression and the successes of available interventions can guide strategies to mitigate “chemobrain,” thereby improving the quality of life for cancer survivors.

#### 3.2.2. Dox-Induced Structural and Morphological Changes to Brain Tissue

Beyond impairing neurogenesis, Dox treatment causes profound structural and morphological changes to brain tissue, which can be visualized through histopathological analyses. Histopathological studies have shown that Dox exposure results in abnormal dendritic development, disrupted neuronal organization and shape in the cortex and hippocampus, and increased neuronal degeneration [101,108]. Dox-induced neuronal damage was particularly evident through a reduction in cell number, increased autolysis and necrosis, disordered arrangements of nerve cells with edema—excess fluid build-up—, swelling, and deformation [114,115,116]. Type II Herring bodies, characteristic of degenerated neurosecretory axons, were observed in circumventricular organs [116].

Moreover, Dox-treatment in mice increased glial fibrillary acidic protein, an astrocyte biomarker, in the CA3 region of the hippocampus which correlated with reactive astrogliosis. Reactive astrogliosis is the production of astrocytes in response to CNS damage and is necessary for the onset and progression of many neuropathies [64,86,117]. Given the important role of the CA3 region in pattern separation and pattern completion, damage to this area would further help explain memory impairment in patients treated with Dox [118]. Physical exercise such as running improves memory in addition to its ability to enhance immature neuron proliferation in the hippocampus [119,120].

Damage is not restricted to the hippocampus—Dox also induces morphological alterations in the cerebral cortex, which governs key executive functions such as problem-solving, thinking, and planning. The cerebral cortex’s six layers, composed of granule, pyramidal, stellate, and fusiform cells, showed marked gliosis, a spongiform appearance of brain tissue, swollen mitochondria, and a dilated endoplasmic reticulum (ER) following Dox treatment [101]. Shrunken pyramidal and granular cells with pyknotic nuclei—shrunken and irregular nuclei commonly formed during necrosis or apoptosis—were also observed [114,115,121]. In line with this, Mohamad et al. revealed significantly increased apoptosis in the cortex of Dox-treated rats using immunofluorescence staining [121].

Importantly, co-administration with neuroprotective agents like luteolin and QE alleviated these histopathological and ultrastructure changes, increasing neuronal cell density in the cerebellum, motor cortex, and hypothalamus, which is indicative of improved neuronal health in regions critical for motor and cognitive function [74,101]. Similarly, treatment with DPDS preserved neuronal structure and health in the cerebrum and cerebellum, as evidenced by increased Nissl substance intensity, a marker of healthy neurons [75]. Disruption of nascent neuron maturation and morphology, characteristic of brain injury in the hippocampus and the cortex, points to disrupted cognitive function upon Dox exposure. Animal models with these deficits display impaired hippocampal-dependent memory tasks, such as poor contextual fear memory, but intact amygdala-dependent cue-specific fear memory, deficits in reference spatial memory, and poor novel object recognition [108,110,122].

### 3.3. Impaired Neurotransmitter Regulation

Neurotransmitter synthesis, release, uptake, and receptor activity are crucial for maintaining proper function of the neural circuit and overall cognitive health. Patients undergoing Dox treatment show dysregulation of these processes, leading to impaired long-term potentiation (LTP) and synaptic plasticity, a process critical for learning and memory [123]. Deficits in learning and memory ultimately affect neurotransmitter regulation and synaptic integrity, targeting the mechanisms contributing to cognitive decline and behavioural changes.

#### 3.3.1. Dysregulation of Neurotransmitter Systems

LTP is a process by which synaptic connections are strengthened over time, forming the cellular basis for learning and memory. This process depends on the proper function of both presynaptic and postsynaptic mechanisms and relies heavily on the proper function of key glutamate receptors that mediate excitatory neurotransmission. LTP is also a common target of Dox treatment characterized by the disruption of neurotransmitter pathways and receptor function. N-methyl-d-aspartate receptor (NMDAR) and α-amino-3-hydroxyl-5-methyl-4-isoxazole-propionate receptor (AMPAR) are glutamate receptors required for the induction of LTP and excitatory neurotransmission [28,110]. Glutamate is a primary excitatory neurotransmitter found throughout the CNS [124]. A study reported that Dox treatment decreased AMPAR function and downstream protein signalling, including brain-derived neurotrophic factors in the hippocampus of mice, which can reduce Ca^2+^ influx at synaptic terminals [110]. On the other hand, another study reported increased expression of AMPAR and NMDAR subunits that were permeable to Ca^2+^ upon Dox treatment [123]. Ca^2+^ homeostasis is essential for neurotransmission and neuronal excitability. Ca^2+^ acts as a crucial intracellular messenger in neurons, regulating a wide range of processes from neurotransmitter release to synaptic plasticity. Reduced Ca^2+^ prevents LTP and impairs synaptic plasticity and spatial memory, whereas increased Ca^2+^ influx over-activates neurons and leads to neuron toxicity (excitotoxicity) [110,123]. In fact, increased Ca^2+^ influx could help explain the opening of the mPTP since Ca^2+^ binds to cyclophilin D, which is a key regulatory protein of the mPTP [125]. This toxicity can start a cascade of deleterious effects that cause oxidative stress and mitochondrial dysfunction, which contribute to further neuronal damage and indirectly cause neuronal death [126]. Such Ca^2+^-associated learning and memory deficits are also found in the aging brain, which highlights the complex ways in which DIN can affect cognitive function and neurodegeneration [127].

The neurotransmitter glutamate requires precise regulation due to its crucial role in preventing excitotoxicity: a condition where accumulated glutamate levels overstimulate neurons, eventually leading to cell death [128]. Thomas et al. reported reduced glutamate clearance by 45% and 48% in the frontal cortex and dentate gyrus of Dox-treated mice, respectively, and increased potassium (K^+^)-evoked glutamate overflow in the dentate gyrus [129]. These changes may contribute to excitotoxicity and synaptic dysfunction, further exacerbating cognitive deficits observed following Dox treatment. Excessive glutamate levels in humans are cleared by astrocytes using excitatory amino acid transporters (EAATs) in the synaptic cleft [130]. EAAT hypo-expression or hypofunction is implicated in many neurodegenerative diseases [131]. TNF-α was found to inhibit EAAT2; thus, Dox-induced inflammation (discussed in the next section) can explain the cognitive implications of DIN which are like those seen in patients with chronic neurodegenerative diseases [132]. Moreover, EAAT dysfunction prevents cysteine uptake into neurons which is required to produce GSH and mitigate oxidative stress [133]. Recent studies aimed to explore therapeutic strategies that restored glutamate homeostasis by targeting the antioxidant NAC [134]. NAC can indirectly modulate extracellular glutamate levels via increasing the exchange between cysteine uptake and glutamate export across EAAT3, releasing more glutamate into the extracellular space [133]. EAAT3 has a higher affinity for cysteine than other EAATs; thus, NAC-mediated elevated function of EAAT2 may lead to increased uptake of extracellular glutamate from the synaptic cleft, allowing for a reduction in glutamate concentration and preventing the excitotoxity normally induced by Dox [135].

The enzyme acetylcholinesterase (AChE) regulates acetylcholine (ACh) levels in the synaptic cleft. ACh is a major neurotransmitter in the CNS and PNS crucial for memory function, coordinating neuronal network responses, and setting the cholinergic tone [136]. ACh turnover by AChE terminates the actions of the neurotransmitter, avoids constant activation of cholinergic receptors, and allows for precise control over synaptic transmission. Beyond its classical role of breaking down ACh, AChE also engages in non-classical signalling pathways (non-hydrolytic activity) that influence neuronal excitability and survival. Dysfunction of AChE has also been implicated in DIN. For example, Dox administration significantly increased hippocampal AChE expression and activity, which disrupts cholinergic signalling critical for learning and memory. On the other hand, some animal models reveal that Dox treatment downregulated AChE activity, which led to excessive ACh accumulation and resulted in excitotoxicity [136,137,138]. Interestingly, a non-classical function of AChE has been linked to similar outcomes as those associated with cancer and neurodegeneration, where the AChE-derived C-terminal peptide acts at an allosteric site on the α7 nicotinic acetylcholine receptor (α7-nAChR) instead of ACh, thereby enhancing Ca^2+^ influx [139,140,141]. Dox-induced AChE dysfunction may exacerbate this response, making it relevant to regulate the classical function of AChE. Co-administration with antioxidants such as astaxanthin and luteolin normalized AChE activity in the cerebellum, cerebrum, and hypothalamus of Dox-treated rats, restored hippocampal architecture, and alleviated associated memory deficits [74,137]. Additionally, co-treatment with AChE inhibitors donepezil and galantamine improved spatial learning, memory retention, and behavioural outcomes in Dox-treated animals, highlighting the therapeutic potential of targeting cholinergic pathways to mitigate Dox-induced cognitive dysfunction [28,142].

Finally, monoamine neurotransmitter systems are disrupted following Dox administration. Monoamines, including dopamine, serotonin, and norepinephrine, are crucial for mood regulation, attention, and executive function. These neurotransmitters are damaged by Dox-induced ROS, which leads to decreased monoamine activity in the CNS and PNS, reflecting widespread neurochemical dysregulation [143]. Dox-induced inhibition of ATP production also prevents monoamine synthesis, resulting in decreased storage and release of essential monoamines [96]. Treatments such as naringin, a compound with neuroprotective properties, and sertraline, a selective inhibitor of serotonin uptake and a weak inhibitor of dopamine and norepinephrine uptake, have shown efficacy in restoring monoamine levels, improving behavioural outcomes, and mitigating anxiety-like and depressive-like symptoms associated with DIN [96]. Neurotransmitter levels need to be maintained at basal levels as even high neurotransmitter levels can lead to cellular dysfunction. For example, the activity of monoamine oxidase (MOA), an enzyme required for regular brain monoamine neurotransmitter turnover and therefore normal synaptic transmission, was inhibited by Dox. This was linked to elevated dopamine levels in the cortex and hippocampus of rats, which led to oxidative stress and disrupted prefrontal cortex function. Co-treatment with nanocurcumin brought dopamine back to basal levels, increased MAO activity, and was associated with reduced oxidative stress [138].

#### 3.3.2. Synaptic Dysplasia

The structure and stability of synaptic connections rely heavily on cytoskeletal and scaffold proteins, which organize synaptic components and support effective neurotransmission. Synaptic dysplasia is heavily associated with deficits in synaptic structure and function. Structural changes include altered morphology in the dendritic spine, reduced spine density, and impaired synaptic architecture, while functional impairments are characterized by disrupted NT release, receptor function, and synaptic signalling. Acute Dox exposure impaired LTP, which is indicative of compromised synaptic plasticity [85]. In fact, synaptic dysplasia is a well-known hallmark for DIN. Dox treatment induces synaptic dysplasia through two major mechanisms, cytoskeletal and scaffold protein disruption and oxidative stress [129]. Of importance is that this disruption of LTP occurs without affecting presynaptic function, suggesting postsynaptic disruptions in signalling [85]. However, a proteomic study in a Dox-treated breast cancer cell line detected downregulation in pathways pertaining to CNS neuron differentiation and the neuron projection membrane [144]. Replication of these findings in Dox-treated neurons would provide another mechanism that may contribute to impaired synapse function.

Disruption of cytoskeletal and scaffold proteins, including synaptophysin and synapsin, led to impaired synaptic architecture maintenance [112,145]. Damage in these proteins caused a loss of dendritic spine and synaptic connections, resulting in synaptic dysplasia, particularly in the hippocampal neurons, vital for learning and memory [146]. In fact, Dox treatment reduced synapsin and synaptophysin expression in vitro and in the cerebral cortex and hippocampus of rats, both of which correlated with neurite loss. Oxidative stress was reported to reduce synaptic function in neurons with altered levels of synaptic markers such as synapsin-1 and postsynaptic density protein 95 in the hippocampus of Dox-treated rats [147]. Oxidative stress is also implicated in hindering LTP, which causes an imbalance between excitatory and inhibitory neurotransmission, aggravating cognitive function impairment [148]. Neuronal impairment associated with reduced synapsin and synaptophysin expression was alleviated by levetiracetam and QE, respectively, showing neuroprotective activity [101,112].

### 3.4. Autophagic Dysregulation

Autophagy, or “self-eating”, is a fundamental cellular process that plays a key role in maintaining cellular health by degrading and recycling damaged or dysfunctional components. Cellular biowaste such as proteins, nucleic acids, lipids, glycogen, and organelles are recycled via lysosomal degradation into metabolic building blocks, such as amino acids, nucleotides, fatty acids, etc., which can be reused for cellular maintenance and energy production [149]. All cells engage in this pathway as it is necessary to maintain cellular homeostasis and to respond to cellular stress [81,149,150]. In particular, the process involves (1) the initiation and formation of a pre-autophagosome, (2) maturation into an autophagosome upon biowaste enclosure, and (3) autophagosome–lysosome fusion and lysosome-mediated biowaste degradation [12,151]. This dynamic process is also known as autophagic flux. Defective autophagy leads to the accumulation of mitochondria and abnormal autophagic substrates, autophagosomes and oxidative stress, and reduced antioxidant enzyme activity [81,152]. In the brain, impaired autophagic flux can accelerate aging, lead to lipid droplet accumulation in neurons, impair cognitive function, and lead to neuronal death [81,153].

#### 3.4.1. Dox-Induced Autophagic Dysregulation in the Brain

Dox impairs autophagic flux at all stages in the pathway. Dox treatment increased levels of neural microtubule-associated protein light chain 3 II (LC3-II) and p62, markers of autophagosome formation and degradation, respectively, in the hippocampus of rats [81,153]. LC3-II is a protein that associates with autophagosomes during their formation, and elevated LC3-II expression can indicate either an increase in autophagosome formation upon Dox exposure or impaired autophagosome–lysosome fusion. Elevated protein levels of the selective autophagic substrate p62, on the other hand, indicate impaired autophagosome degradation [81,153]. Lysosomal proteolytic activity was also impaired due to Dox-induced alkalization of lysosomes [153]. Moreover, p62 participates in multiple critical signalling pathways, including activation of the NRF-2-regulated antioxidant pathway which is critical in protecting neural cells from oxidative injury [154]. In fact, Dox reduces cytoplasmic and nuclear NRF-2 protein expression, thus increasing oxidative stress in the nervous system. NRF-2’s antioxidant function was rescued upon pretreatment of rats with antioxidant curcumin with known, anti-inflammatory, and neuroprotective functions [154]. Dox also affects Beclin-1 expression, a protein essential for the formation of pre-autophagosome [81,153].

#### 3.4.2. Prospective Therapeutic Interventions

It has been proposed that upregulating transcription factor EB (TFEB) may be neuroprotective in patients with Dox-mediated impaired autophagy due to its role in promoting autophagosome degradation and preventing dysfunctional organelle accumulation [153]. Overexpression of TFEB in Dox-treated neurons normalized autophagic flux through improved lysosomal acidity and improved neuronal survival by restoring cellular homeostasis [153]. Co-treatment of Dox-exposed neurons with a pharmacologic activator of TFEB improved autophagy and survival [153,155]. Altogether, it is evident that Dox impairs autophagic flux in neural cells and contributes to neurotoxicity, and that restoring autophagy is crucial to prevent the adverse impacts of autophagy dysregulation on DIN and to improve neural cell survival.

## 4. Mechanisms of Indirect DIN

**Figure 5:**
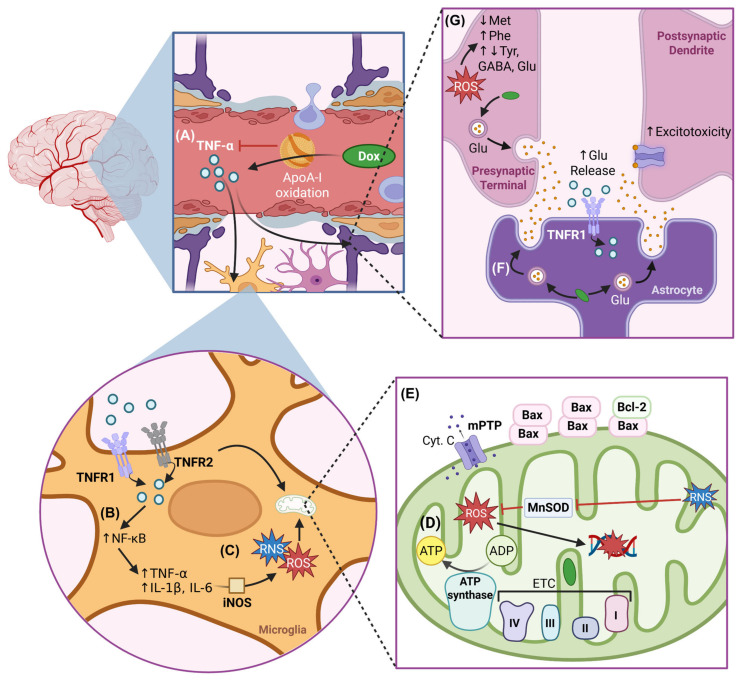
A schematic of the mechanisms of indirect DIN. A large majority of DIN is induced by the indirect effects of Dox on brain tissue. (**A**) Dox triggers peripheral TNF-α production via the oxidation of ApoA-I. The inflammatory molecule crosses the BBB and enters microglia via TNFR1 and 2 and astrocytes via TNFR1, thereby activating both glial cells. (**B**) Microglia activation triggers inflammation due to the production of inflammatory signalling molecules and cytokines such as NF-kB, IL-1β, and IL-16. This induces (**C**) oxidative stress through ROS and RNS production by iNOS. The ROS/RNS then go and trigger (**D**) mitochondrial dysfunction and (**E**) neural apoptosis via the same mechanisms as they did in direct DIN. (**F**) Astrocyte activation induces neurotransmitter dysfunction via increased glutamate release into the synaptic cleft as it leads to excitotoxicity. (**G**) Finally, elevated ROS impair amino acid (Met, Phe, Tyr, Glu, GABA) turnover, which is required for neurotransmitter synthesis, contributing to metabolic dysfunction. ApoA-I: apolipoprotein A-I; DIN: Dox-induced neurotoxicity; BBB: blood–brain barrier; Dox: doxorubicin; GABA: gamma-aminobutyric acid; Glu: glutamine; IL-1β: interleukin-1 beta; IL-6: interleukin-6; iNOS: inducible nitric oxide synthase; Met: methionine; MnSOD: manganese-containing superoxide dismutase; mPTP: mitochondrial permeability transition pore; NF-κB: nuclear factor kappa beta; Phe: phenylalanine; ROS: reactive oxygen species; RNS: reactive nitrogen species; TNF-α: tumour necrosis factor-alpha; TNFR1/2: tumour necrosis factor-alpha receptor 1/2; Tyr: tyrosine.

Due to the restricted permeability of the BBB, the majority of Dox-induced CNS toxicity occurs indirectly through systemic pathways. Dox-induced oxidative stress-mediated elevation of pro-inflammatory cytokines and their ability to cross the BBB is proposed as the leading mechanism of indirect DIN [96,150]. Dox-induced ROS upregulate systemic inflammatory cytokines that cross the BBB and ultimately impact neurogenesis, survival, neuronal viability, and other factors contributing to DIN [21]. A schematic overview of the mechanisms involved in indirect DIN can be found in Figure 5.

### 4.1. Neuroinflammation

Oxidation-mediated neurotoxic effects of Dox are compounded by neuroinflammation via elevated TNF-α levels, which can cross the BBB via receptor-mediated transport and activate microglia and astrocytes [21,156,157]. Astrocytic TNF receptor-mediated TNF-α signalling is associated with increased glutamate release at synapses in the hippocampal dentate gyrus, indicating enhanced presynaptic activity [156]. Dox-mediated elevated TNF-α can similarly impair synaptic activity observed in Dox-treated animals. In addition, microglial activation triggers the local production of additional TNF-α and pro-inflammatory cytokines, such as interleukin-1 beta (IL-1β) and IL-6 via NF-κB activity [21,70,83]. These cytokines exacerbate oxidative stress by stimulating the production of additional ROS and RNS via iNOS, ultimately perpetuating a cycle of neuroinflammation and oxidative stress [13,83,86,90]. Elevated TNF-α in hippocampal tissues correlates with increased apoptosis, highlighting the role of inflammation in aggravating neuronal damage in DIN [28,72,83,123]. These inflammatory changes contribute to the development of anxiety and depression-like behaviours and underscore the multifaceted nature of DIN.

Studies have demonstrated that neutralizing systemic TNF-α reduced brain mitochondrial injury, oxidative damage, and neuronal apoptosis, highlighting its pivotal role in the inflammatory cascade [76,83,90]. In TNF-*α* KO (TNFKO) mice, Dox-induced oxidative stress was significantly mitigated, and mitochondrial respiration was preserved. Neurochemical disruption in the hippocampus, such as reduced choline/creatine ratios observed in wild-type mice, was partially restored in TNFKO mice treated with Dox, demonstrating the neuroprotective effect of TNF-*α* deficiency [69]. Additionally, antioxidant supplementation with 2-mercaptoethane sulfonate sodium (MESNA), ω-3 PUFAs, QE, xanthone, galantamine, and dl-NBP has been shown to reduce neuroinflammation by downregulating inflammatory mediators (TNF-α, NF-κB, IL-1β, lipoxygenase, and cyclooxygenase) [28,70,72,80,100,101].

### 4.2. Impaired Metabolism

Dox’s systemic impacts lead to profound alterations in brain metabolism. These changes impact key metabolic pathways that regulate essential functions such as cholesterol, amino acid and energy metabolism, and neurotransmitter regulation. These metabolic disruptions can contribute to DIN by compromising the proper functioning of neuronal cells and impairing cognitive functions.

#### 4.2.1. Impaired Cholesterol Metabolism and Apolipoprotein A-I Dysfunction

Dox-mediated lipid peroxidation produces reactive aldehydes like HNE which form adducts with critical proteins such as apolipoprotein A-I (ApoA-I), impairing its structure and neuronal membrane function [158]. ApoA-I is integral to regulating TNF-α-mediated inflammatory responses and to reverse cholesterol transport, a process critical for maintaining lipid balance in the brain, whereas cholesterol is essential for maintaining neuronal membrane fluidity, synaptic plasticity via myelin integrity, and neurotransmitter receptor function [159,160]. Oxidized ApoA-I is unable to suppress serum TNF-α, exacerbating the oxidation-mediated inflammatory responses [90,158,161]. Interestingly, oxidative damage inflicted to ApoA-I by the peroxidase myeloperoxidase reduced its interaction with ATP-binding cassette transporter A1 (ABCA1), a key protein mediating cholesterol efflux [162]. Dox-induced oxidative damage and resultant ApoA-I adducts may similarly disrupt ABCA1 interaction and compromise cholesterol homeostasis. This could lead to cholesterol accumulation in neuronal membranes and affect neuronal membrane repair and synaptic plasticity [162]. The observed cholesterol elevation upon Dox treatment has widespread impacts as it occurs in multiple tissues apart from the brain, namely the heart, liver, and kidney [159]. Dox impairs neuronal membrane health by decreasing phosphatidylcholine-specific phospholipase D activity, an enzyme critical for maintaining membrane health [90]. The associated upregulated neuroinflammation exacerbates metabolic dysfunctions by reducing complex I activity and amplifying lipid peroxidation [69].

#### 4.2.2. Impaired Amino Acid Metabolism

Metabolomic studies revealed that Dox induced region-specific changes in amino acid and lipid metabolism in the brain. Altered amino acid levels are highly associated with Dox-induced oxidative damage [163]. Key alterations included reductions in methionine and elevated phenylalanine—precursors for neurotransmitter dopamine—and altered tyrosine, glutamate, and GABA levels in the hippocampus, prefrontal cortex, and neocortex, which impairs neurotransmitter synthesis [164]. Moreover, disrupted GABA and glutamate balance contributes to excitotoxicity [164,165]. On the other hand, elevated phenylalanine and reduced methionine reflect molecular imbalances observed in aging mouse brains [164]. Correlations between amino acid level alterations in the brain to their levels in the liver and kidney suggest that Dox-induced systemic metabolic disorders indirectly affect brain function [159].

Therapeutic strategies aimed at restoring metabolic balance and enhancing antioxidant defenses offer promise for mitigating DIN. Antioxidants such as ALA and naringin have shown efficacy in reducing ROS levels and inflammation, restoring mitochondrial function and antioxidant levels, and improving cell viability [163,166]. ALA exerts these antioxidant effects via the NRF-2 signalling pathway [163]. Naringin prevented an imbalance in lipid metabolism by restoring levels of urea, cholesterol, triglyceride, and creatine kinase that are normally elevated by Dox [166]. Understanding the interplay between these metabolic pathways provides a foundation for developing targeted therapies to protect the brain during Dox-based chemotherapy.

Table 1 provides a summary of the main proteins involved in the aforementioned direct and indirect DIN mechanisms, as well as the outcomes induced by Dox and the relevant studies.

## 5. Endotheliotoxicity and BRCA1/2 in Cancer and Neurotoxicity

### 5.1. Endotheliotoxicity

The BBB is composed of BMECs that mediate signalling between the systemic circulation and the CNS to support neuronal metabolism, which is vital for optimal functioning of the brain’s ~86 billion neurons [167]. Endothelial cell damage upon exposure to physical and chemical stresses disrupts key endothelial functions such as its role in the formation of a protective barrier. This leakiness facilitates the passage of unwanted substances from the circulation into the brain. Prolonged endothelial dysfunction and apoptosis, coined endotheliotoxicity, can impair the BBB and lead to neuronal and brain damage. Virtually all cytotoxic pathways that affect neurons and glial cells upon Dox exposure also impact endothelial cells. Therefore, it is imperative to obtain a comprehensive understanding of the mechanisms that alter endothelial function and lead to DIN.

#### 5.1.1. Endothelial Dysfunction

The endothelium is a dynamic, multifunctional layer of cells lining blood vessels that plays a crucial role in vascular homeostasis. Under physiological conditions, endothelial cells produce nitric oxide via endothelial NOS (eNOS) which promotes vasodilation, inhibits platelet aggregation, and suppresses inflammation [168]. Another critical function is immune regulation, where endothelial cells, in a resting state, prevent excessive leukocyte adhesion and migration [169]. However, under pathological conditions, these finely tuned mechanisms become dysregulated, leading to endothelial dysfunction, a hallmark of cardiovascular diseases and neurodegeneration. Endothelial dysfunction is primarily characterized by a loss of NO bioavailability, which impairs vasodilation and promotes vascular stiffness and inflammation. A key driver of this dysfunction is oxidative stress, which contributes to reversible endothelial activation where endothelial cells enter a pro-inflammatory, procoagulant, permeable, and proliferative state [170,171]. Endothelial cells express adhesion molecules such as ICAM-1, VCAM-1 and E-selectin, facilitating leukocyte adhesion, infiltration, and chronic inflammation. Activation of inflammatory pathways further amplify endothelial dysfunction by upregulating pro-inflammatory cytokines [167]. Over time, these pathological changes become irreversible, driving non-compensatory vascular remodeling and contributing to diseases [167,170].

Extending beyond the systemic vasculature, endothelial dysfunction significantly impacts the BBB. In aging and neurodegenerative diseases, BBB integrity is compromised, contributing to increased permeability, disrupted homeostasis, neuronal injury, and cognitive decline [169]. Oxidative stress and inflammation primarily disrupt the BBB, leading to the expression of adhesion molecules and the recruitment of immune cells, which can further damage the BBB [167,169]. Additionally, pericyte loss and astrocyte dysfunction, often observed in aging, exacerbate BBB permeability and contribute to neurodegenerative processes.

#### 5.1.2. Dox-Induced Endothelial Dysfunction, Endotheliotoxicity, and Neurotoxicity

Dox’s cytotoxic mechanisms impair key elements of endothelial function and promote endotheliotoxicity. While limited studies exist that assess the direct impact of Dox on endothelial cells composing the BBB, there are many findings pertaining to the general correlation between Dox treatment and endotheliotoxicty which can be extrapolated to BMEC toxicity. This is of importance as Dox-induced endothelial damage can compromise BBB integrity, leading to increased permeability and subsequent neuronal exposure to neurotoxic substances [32,33,34,35].

Dox-induced oxidative stress plays a pivotal role in endothelial dysfunction. Specifically, ROS overproduction disrupts the bioactivity of eNOS and endothelin-1, which reduces nitric oxide bioavailability, leading to altered vascular tone and RNS-mediated oxidative stress [20,35,172]. Many of these findings were obtained in cultured human endothelial cells, further supporting the notion that a similar mechanism of oxidative damage may occur in BMECs. Interestingly, restoration of cardiac NO preserved cardiac function in Dox-treated mice [173]. Dox-induced oxidative damage eventually leads to endothelial cell apoptosis and endothelium permeability. Tight junction proteins like ZO-1 are crucial to keep the endothelium, including the BBB, impermeable, thereby protecting tissues such as neurons from harmful substances. Dox has been observed to inhibit ZO-1 expression in endothelial cells, resulting in an increased endocardium permeability to Dox [35]. Furthermore, Dox-induced mitochondrial dysfunction in endothelial cells can lead to energy deficits and greater ROS production, which may affect neural cells that rely on proper endothelial function for nutrient and oxygen supply [172]. The release of cytochrome c and subsequent activation of apoptotic pathways in endothelial cells can also have downstream effects on neuronal survival.

Endothelial cells express both isoforms of Topo II (IIα and IIβ) due to their proliferative nature, especially during stress-induced activation, wound healing, and angiogenesis [35]. This characteristic renders them susceptible to Dox-mediated inhibition of Topo II, as well as Dox-induced DNA intercalation and micronuclei formation. The presence of cytoplasmic micronuclei activates the cGAS-STING pathway, leading to the production of IFN-α/β, which contributes to chronic endothelial inflammation and damage [35,174]. Moreover, Dox-induced cellular stress and endothelial cell activation further promote inflammatory responses in endothelial cells through activation of the NF-κB pathway [175]. In the brain, these inflammatory cytokines can further damage the BBB and promote infiltration of peripheral inflammatory mediators and immune cells into the CNS, exacerbating inflammatory injury in neural tissues.

Autophagy is critical for endothelial function and its metabolic processes; as such, impaired autophagy leads to endothelial dysfunction. Since Dox impairs autophagy at various stages in neurons, it is likely that a similar mechanism of dysregulation occurs in endothelial cells, pointing to Dox-induced endothelial dysfunction via impaired endothelial autophagy. A recent study reported an increased LC3II/LC3I ratio in endothelial cells upon Dox treatment even in the presence of the autophagy inhibitor, pointing to upregulated autophagy. While the effects of Dox on essential autophagy-related proteins (ATG and p62) were not measured, the authors found that Dox inhibited mTOR, sustaining autophagy, and suppressed VEGFR2, a receptor for VEGF-α which regulates endothelial function and autophagy [176]. Furthermore, endothelial cell-specific loss of autophagy was shown to exacerbate Dox-induced mortality [177]. These findings reinforce that Dox induces endothelial dysfunction and impairs endothelial autophagy. Similarly, it is likely that Dox-induced endothelial autophagy impairment compromises the BBB and contributes to DIN. Emerging evidence also suggests that Dox can induce the endothelial-to-mesenchymal transition, a process where endothelial cells lose their function, exacerbating endothelial dysfunction [178].

Despite the significant role of Dox in inducing endotheliotoxicity and the importance of endothelial health in cognitive function, there remains a lack of investigation on Dox-induced endotheliotoxicity in the BBB and associated neurotoxicity. Although Dox’s ability to cross the BBB is limited, endothelial cells are one of the first cell types to interact with circulatory Dox, enabling the drug to induce oxidative and nitrosative stress on the BBB. We propose endothelial dysfunction as a novel mechanism contributing to DIN, warranting further investigation.

### 5.2. DDR Impairments, Cancer, and Neurodegeneration

As discussed, defective BRCA1 and BRCA2 increase cancer risk in men and women due to increased genomic instability [14,18]. However, non-functional checkpoint control is required in combination with impaired genome integrity for the development of cancer as defective checkpoints enable damaged cells to continue proliferation. In fact, common genetic alterations associated with BRCA mutants include a loss of ATM expression and mutations in *TP53* (the gene encoding p53) [14]. Given the vast range of roles that BRCA1 plays in checkpoint activation, mutated BRCA1 could also impair checkpoint control throughout the cell cycle. Moreover, the broader range of functions of BRCA1 implies that it is the more critical counterpart of the two BRCA genes, with more severe genotoxicity found in tumours associated with faulty BRCA1 than defective BRCA2 [14,179]. In line with this, women carrying BRCA1 mutations have a higher risk of developing breast and ovarian cancers [180,181]. In contrast, BRCA2 mutations are associated with an increased risk of developing cancers of the breast, prostate, pancreas, and skin in men [182,183].

Additionally, BRCA mutation carriers were found to be at a higher risk of cardiovascular disease and neurodegeneration [57]. In fact, over half of breast cancer deaths are because of organ dysfunction-related complications [184]. This is because defects in DDR in proliferating cells can lead to cancer, while the same defects in post-mitotic cells such as adult cardiomyocytes and neurons lead to cardiomyopathy and neurodegeneration, respectively [24,185]. Surprisingly, nucleotide excision repair was found to be repressed in terminally differentiated cells. Being terminally differentiated, neurons would be under relatively more stress due to a second attenuated DDR mechanism [186,187]. Knockdown of BRCA1 led to changes in neuronal structure that impaired LTP in humans. BRCA1 loss was also associated with learning and memory deficits like “chemobrain” symptoms. In mature, non-mitotic neurons, BRCA1 reduction did not impair cognitive function through neuron apoptosis, meaning that the negative impacts of BRCA1 deficiency are context-dependent [188]. A preclinical study in 2014 revealed a novel interaction and feedback mechanism between BRCA1 and sirtuin 1 (SIRT1), an NAD-dependent histone deacetylase that is linked to metabolism, ER stress responses, epigenetic regulation, and genome stability. The authors proposed a compensatory mechanism for maintaining SIRT1-related functions that could provide therapeutic options for BRCA1-related ovarian cancers in the context of ER stress resistance [189]. This is of importance as sustained ER stress, due to attenuated ER stress response, initiates apoptosis and is linked to neurodegenerative diseases, whereas hyperactivation of this stress response mechanism is observed in cancer [190].

On the other hand, a study proposed BRCA1 as a potential early biomarker for AD since BRCA1 dysfunction correlated with DNA fragmentation, genomic instability, and synaptic loss, all hallmarks of AD [191]. In fact, BRCA1 hyperactivation, despite having a protective role initially, has been found to trigger proapoptotic pathways depending on the neurodevelopmental stage of neurons [188,191]. Additionally, overexpression of the BRCA1-Δex11 isoform, which lacks a nuclear localization signal, is particularly relevant in pathological conditions and upon increased DDR induced by Dox [192]. BRCA1-Δex11 overexpression and its mis-localization contributes to cell cycle deregulation, centrosome dysfunction, and apoptosis in AD brains [191]. To further support these findings, post-mortem studies show hypomethylation of the BRCA1 promoter in AD brains, leading to increased BRCA1 expression and cytoplasmic mis-localization [193]. Thus, the isoform and subcellular location of BRCA1 determines whether the DDR protein has a protective or pro-death role. This opens targeting BRCA1-related pathways as a viable therapeutic intervention for neurodegenerative diseases.

There is considerable evidence in the literature pointing to a connection between overall impaired DDR and neurodegeneration. For example, p53 dysfunction-associated events are linked to chronic neurodegenerative diseases including AD [194,195,196], PD [197,198], HD [199], Down syndrome [200,201], ALS [202,203], and MS [204,205]. Dietary supplements can increase tumour suppressor (BRCA1 or p53) activity or remove hypermethylation on tumour suppressor genes to restore their expression and in turn mitigate neurodegeneration associated with AD [196]. Such interventions may prove to be beneficial in ameliorating “chemobrain” symptoms in anthracycline treatment cancer patients.

Many cancer patients have been characterized by increased neurodegeneration [197,206,207,208,209,210] and recent studies have also revealed the role of miRNAs, protooncogenes (DJ-1), and other tumour suppressor genes (MM-1) in the pathology of both diseases [211,212]. Unfortunately, the correlation between cancer and neurodegeneration negatively impacts the quality of care that patients with cognitive impairments (such as dementia) receive. This includes late diagnosis, less treatment yet more invasive procedures, more treatment complications, and poorer survival compared to cancer patients without dementia [213,214]. Evidently, there is a need for standardized guidelines for decision-making and eventual cancer treatment in patients with dementia to avoid biases.

**Figure 6:**
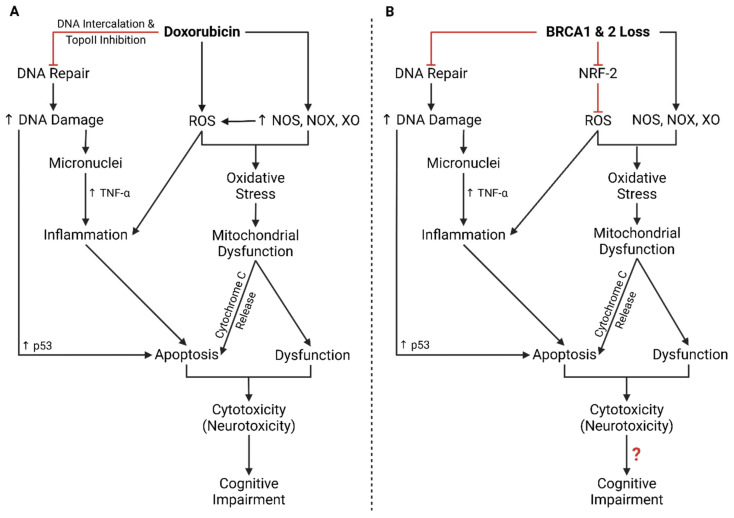
A comparison between (**A**) doxorubicin’s mechanism of cytotoxicity in the brain and (**B**) the impact of a loss of BRCA1 or 2 functions. (**A**) Dox induces oxidative stress in cells via the upregulation of NOS, NOX, and XO and the overproduction of ROS. The generated oxidative stress leads to mitochondrial dysfunction and overall cellular dysfunction and apoptosis, triggered by cytochrome c release. At the same time, Dox intercalates with DNA and inhibits Topo II, impairing DNA repair while exacerbating DNA damage. DNA damage leads to inflammation via micronuclei- and TNF-α-mediated signalling, ultimately leading to cell death. Cytotoxicity in neurons (neurotoxicity) induces cognitive impairment. (**B**) BRCA1 and 2 loss follows a similar mechanism, with impaired DNA repair and dysfunctional antioxidant activity (NRF-2) being direct consequences. BRCA loss-mediated neurotoxicity may lead to cognitive impairment, however, research on this relationship is still emerging and is therefore represented with a “**?**”. BRCA: breast cancer gene; NOS: nitric oxide synthase; NOX: NADPH oxidase; NRF-2: nuclear factor erythroid 2-related factor 2; ROS: reactive oxygen species; RNS: reactive nitrogen species; Topo II: Topoisomerase II; TNFα: tumour necrosis factor-alpha; XO: xanthine oxidase.

A loss of BRCA1 and 2 due to haploinsufficiency or heterozygous mutations has similar effects to that of Dox treatment (Figure 6). Overlap in the impaired mechanisms suggests that the mechanisms of Dox cytotoxicity may conflict with BRCA1- and 2-regulated pathways. Despite this overlap, there is still a critical gap in knowledge pertaining to BRCA-Dox interactions.

### 5.3. BRCA Mutation Screening, Doxorubicin, and Neurotoxicity

Despite the well-established critical roles that BRCA1 and BRCA2 play in maintaining genomic integrity and determining cellular survival, and the widespread cytotoxic impacts of Dox, there remains an extensive gap in the field pertaining to the effects of BRCA1 and BRCA2 on endotheliotoxicity and DIN.

It should be noted that current genetic screenings for BRCA mutants and HBOC syndrome only look for exonal mutations and do not measure BRCA haploinsufficiency, where haploinsufficiency refers to below-basal BRCA expression or malfunctional BRCA-related phenotypes. As a result, non-neoplastic implications for BRCA haploinsufficiency are overlooked. Notably, factors independent of BRCA mutation such as hormonal regulation, transcription factors, genetic modifiers, and SNPs in promoter/5′ UTR can cause haploinsufficiency. Diagnostic screenings that evaluate Dox-induced cytotoxicity do not consider BRCA haploinsufficient patients as BRCA mutation carriers even though haploinsufficiency is associated with the development of breast and ovarian cancer. Mutations in HDR-associated genes that interact with BRCA—PALB2 [215]; ATM [216,217]; RAD51C/D [218]; BRIP1 [219]; BARD1 [220]; and CHK2 [221]—create an HDR-deficient phenotype and contribute to HBOC syndrome, yet they too are not considered BRCA-related risks of breast cancer [14,222]. In the brain, mutations, mis-localization, and reduced expression of ATM [223], XRCC1 [61,224], BRCC3 [225], p53 [226,227], and FUS [62] also have implications in neurodegeneration, impaired synaptic transmission, and motor dysfunction. As such, these current measures have led to discrepancies in the literature and oversight in clinical examinations for implications of impaired BRCA functions [228,229]. This is reinforced by the fact that the estimated prevalence of pathogenic BRCA mutations in the general population is 1 in 400, with 75% of breast cancer cases associated with non-BRCA genes related to HBOC [230,231]. Due to existing gaps in the literature on the extent of the clinical impact of compromised BRCA genes in patient health, current measures underestimate the effects of BRCA in non-neoplastic settings. Notably, a clinical retrospective study from 2009 observed higher non-cancer mortality associated with BRCA mutation carriers [232].

Apart from haploinsufficiency and HRD, BRCAness is another challenge that needs to be overcome to effectively translate preclinical results to clinical practice. In brief, BRCAness refers to a specific HRD tumour phenotype that mimics the effects of BRCA mutations (e.g., abundant C→T transitions and promoter methylations, rarity of homozygotic variation) without involving actual mutations in the BRCA genes [233]. In fact, BRCAness is prevalent in 20% of breast and 45% of ovarian cancers, and widely present in 21 other cancers. Like BRCA mutants, the BRCAness phenotype is also responsive to poly ADP-ribose polymerase inhibitor (PARPi) treatment, which prevents the repair of single-stranded DNA breaks [233,234]. Additionally, much like haploinsufficiency and HRD, current genetic screenings for HBOC syndrome fail to detect BRCAness. This limitation hinders accurate risk stratification and obscures potential neurotoxic vulnerabilities in patients without canonical BRCA mutations. Effective translation of preclinical results thus requires refined diagnostic strategies that can differentiate between BRCA-mutant and BRCA-associated genotypes and BRCAness phenotypes. Only through such stratification can therapeutic insights from models accurately inform personalized interventions in clinical oncology.

### 5.4. BRCA Mutations in DIN and Endotheliotoxicity

Given the important role of BRCA1 and 2 in maintaining neuronal health, we propose that a loss of expression of either gene has the potential to severely exacerbate DIN and endotheliotoxicity, which compromises the BBB. Although BRCA1 or BRCA2 KO models have primarily been studied in the context of DIC, their findings provide valuable insights into potential neurotoxic mechanisms. Studies demonstrate that cardiomyocyte-specific BRCA1 or BRCA2 deficiency exacerbate DNA DSBs and upregulate mitochondrial dysfunction and apoptotic signalling [17,18]. Given that both cardiomyocytes and neurons are terminally differentiated cells, these findings in cardiomyocytes suggest that neurons lacking BRCA may experience similar vulnerabilities under Dox treatment.

Research is beginning to demonstrate the essential roles of BRCA genes in endothelial function. For example, endothelial cell-specific loss of BRCA2 exacerbated oxidative stress-induced DNA damage, apoptosis, and endothelial dysfunction [235]. Endothelial cell-specific loss of BRCA1 has been found to similarly increase endothelial cell susceptibility to oxidative and inflammatory damage. On the other hand, overexpression of BRCA1 rescued endothelial cells from genotoxic and inflammatory stressors [16]. Combined, this points to a protective role of BRCA genes in endothelial cell function, suggesting that dysfunction of these key DDR genes can impair endothelial health. As these findings were observed in cultured human endothelial cells, it is likely that a loss of BRCA1 or BRCA2 will similarly impair BBB integrity, facilitating Dox passage into the CNS. Interestingly, in our Dox-treated endothelial cell-specific BRCA2 knockout mice, Dox treatment led to an accumulation of Dox, impaired neurotransmitter expression and distribution, and increased DNA damage in brain tissues in comparison to wild-type mice (*own unpublished data*). Another study from the early 2000s reported unchanged brain/plasma concentration ratios of Dox despite treating mice with a drug that impaired P-glycoprotein activity. The results imply that elevated Dox in plasma led to a proportional increase in Dox in the brain, rather than enhanced BBB (endothelium) permeability [27]. However, these experiments were conducted in healthy mice that did not reflect the genetic defects in DDR players commonly found in breast and ovarian cancer patients.

Building on these findings, it is essential to consider how BRCA mutation status may inform prevention or treatment strategies for DIN. Therapeutic interventions differ between BRCA-mutated and non-mutated patient cohorts, with PARPi being a common targeted therapy for BRCA-mutated patients. As mentioned early in this review, there are no treatments for DIN, only preventative strategies to reduced chemotherapy-induced cytotoxicity—such as those discussed in this review— which may be applied to both patient groups. However, PARPi may show promise in mitigating DIN in BRCA patients, as these interventions have shown neuroprotective effects against peripheral nerve injury [236]. Additionally, drugs such as empagliflozin—though not a PARPi—have demonstrated reductions in PARP-1 activity and conferred neuroprotection against CICI in genotypically wild-type rats [237]. Given their ability to prevent DNA repair in rapidly dividing cells, empagliflozin and other BBB-permeable PARPi agents, such as niraparib and pamiparib, offer therapeutic potential for DIN mitigation in BRCA-mutant patients. Future studies should employ endothelium- and neuron-specific BRCA1 or BRCA2 KO models to investigate structural, metabolic, mechanistic, and cognitive changes in the brain under Dox treatment. Expanding on these findings, the inclusion of TNFKO and iNOSKO models in combination with BRCA1 or 2 KOs could provide additional insights into the roles of inflammatory cytokines and oxidative stress in mediating DIN. Examining the interplay between DNA damage repair deficiencies, mitochondrial dysfunction, and inflammatory pathways could uncover novel therapeutic targets for mitigating neuronal damage in breast and ovarian cancer patients.

**Figure 7:**
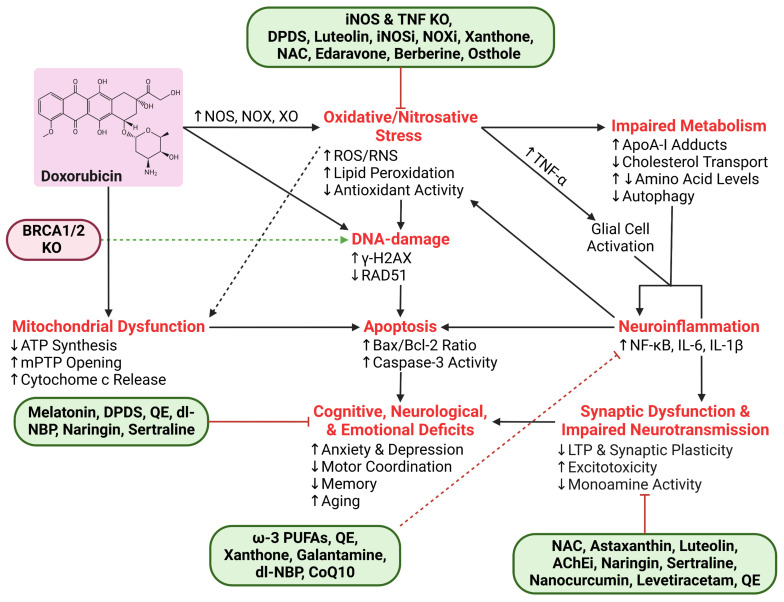
A summary of the mechanistic pathways underlying DIN and available preventative measures. Dox promotes oxidative and nitrosative stress (increased ROS and lipid peroxidation, reduced antioxidants) through NOS, NOX, and XO activity. Concurrently, Dox induces DNA damage such that there is an increase in γH2AX yet a decrease in RAD51-mediated double-stranded break repair. BRCA1/2 KO models display similar DNA damage and trigger the same downstream effects. Dox induces mitochondrial dysfunction both directly and via ROS/RNS, which presents as reduced ATP synthesis and increased mPTP opening-associated cytochrome c release. Accumulating DNA damage and mitochondrial dysfunction leads to endogenous apoptosis activation (increased Bax/Bcl-2 ratio and caspase-3 activity). Oxidative damage impairs metabolism (ApoA-I adducts and impaired cholesterol transport, dysregulated amino acid levels, and impaired autophagy) and induces glial cell activation via upregulation of systemic TNF-α. Both mechanisms induce neuroinflammation, characterized by upregulated pro-inflammatory cytokines (NF-κB, IL-6, IL-1β), along with synaptic dysfunction and impaired neurotransmission (impaired LTP and synaptic plasticity, excitotoxicity, and dysregulated monoamine activity). Neuroinflammation can increase oxidative stress, creating a feedforward loop of oxidative stress and inflammation. Inflammation and neural cell death culminate in cognitive, neurological, and emotional deficits such as elevated anxiety and depression, accelerated aging, and impaired motor coordination and memory. iNOS and TNFKO models and antioxidants like DPDS, luteolin, iNOSi, NOXi, xanthones, NAC, edaravone, berberine, and osthole have been effective in mitigating oxidative stress in Dox-treated systems. ω-3 PUFAS, QE, xanthones, galantamine, dl-NBP, and CoQ10 can successfully mitigate the side effects of Dox-induced neuroinflammation. At the same time, NAC, astaxanthin, luteolin, AChEi, naringin, sertraline, nanocurcumin, levetiracetam, and QE supplementation is effective against Dox-mediated synaptic dysfunction and impaired neurotransmission. Finally, melatonin, DPDS, QE, dl-NBP, naringin, and sertraline are also effective in alleviating Dox-induced cognitive impairments. AChEi: acetylcholinesterase inhibitor; ApoA-I: apolipoprotein A-I; BRCA1/2: breast cancer susceptibility gene 1/2; Co-Q10: coenzyme Q10; dl-NBP: dl-3-n-Butylphthalide; DPDS: diphenyl diselenide; IL-1β: interleukin-1 beta; IL-6: interleukin-6; iNOS: inducible nitric oxide synthase; iNOSi: inducible nitric oxide synthase inhibitor; KO: knockout; LTP: long-term potentiation; MESNA: 2-mercaptoethane sulfonate sodium; mPTP: mitochondrial permeability transition pore; NAC: N-acetylcysteine; NF-κB: nuclear factor kappa beta; NOXi: NADPH oxidase inhibitor; ω-3 PUFAS: omega-3 polyunsaturated fatty acids; ROS: reactive oxygen species; RNS: reactive nitrogen species; TNF-α: tumour necrosis factor-alpha; QE: quercetin.

A summary of the discussed mechanisms of DIN and available interventions can be found in Figure 7.

## 6. Conclusions

In conclusion, Dox is a highly effective yet cytotoxic chemotherapeutic agent, with DIN emerging as a significant but less characterized complication. Dox disrupts neural cell genomic integrity, redox homeostasis, autophagy, and metabolism, which collectively contribute to BBB dysfunction and neuronal injury. Patients treated with Dox commonly experience cognitive impairments including impaired spatial and episodic memory, reduced locomotion, and increased anxiety and depression. Increasing evidence suggests that endotheliotoxicity may play a critical role in DIN, with endothelial dysfunction leading to increased BBB permeability, neuroinflammation, and oxidative stress-related neuronal damage. Furthermore, the impact of Dox on BRCA1 and BRCA2 functions in the CNS remains largely unexplored. Increasing research in this field is imperative as BRCA genes have key roles in DNA repair, the modulation of oxidative stress, and the regulation of cellular stress responses—all mechanisms that are impacted by Dox. Thus, BRCA mutations would negatively affect these key processes and increase Dox-induced cellular stress. Current diagnostic approaches for BRCA mutations overlook haploinsufficiency, thereby missing critical implications of impaired BRCA function in the clinical setting. Emerging studies indicate that BRCA and related DDR genes play essential roles in maintaining endothelial and neuronal function, and their dysregulation may contribute to neurodegenerative complications. Given the noticeable overlap in mechanisms between BRCA-associated phenotypic loss and DIN pathogenesis, further investigation into the functional interplay between Dox and BRCA1 and BRCA2 is warranted. This may include endothelium- and neuron-specific BRCA1 or BRCA2 KO models to investigate structural, cognitive, and mechanistic changes in the brain as well as TNFKO and iNOSKO models to assess the interplay between DDR deficiency, inflammation, and oxidative stress. A deeper understanding of these mechanisms may aid in developing personalized therapeutic strategies for BRCA-mutant cancer patients to mitigate neurotoxicity while preserving the drug’s anticancer efficacy.

## Figures and Tables

**Table 1:** A summary of the different mechanisms of DIN, related proteins and molecules, and their respective studies. AChE: acetylcholinesterase; AMPA: α-amino-3-hydroxyl-5-methyl-4-isoxazole-propionate receptor; ApoA-I: apolipoprotein A-I; Ca^2+^: calcium; DIN: Dox-induced neurotoxicity; Dox: doxorubicin; iNOS: inducible nitric oxide synthase; IL-1β: interleukin-1 beta; IL-6: interleukin-6; LC3-II: light chain 3 II; mPTP: mitochondrial permeability transition pore; NF- κB: nuclear factor kappa beta; NMDA: N-methyl-d-aspartate; NOX: NADPH oxidase; ROS: reactive oxygen species; RNS: reactive nitrogen species; TNF-α: tumour necrosis factor-alpha; XO: X=xanthine oxidase.

Process	Protein/Molecule	Effect of Dox	Study Type
Oxidative Stress	NRF-2	Reduced NRF-2 protein expression and impaired antioxidant functions.	In vivo [154]
	ROS and RNS	Increased production of ROS and RNS via iNOS, XO, and NOX activity.	In vivo [69,71,74,83,86,90] In vitro [68]
Mitochondrial Dysfunction	mPTP	mPTP opening and subsequent cytochrome c release.	In vivo [87]
Neurotransmitter Dysregulation	AMPAR/NMDAR	Altered AMPAR and NMDAR function and Ca^2+^ influx.	In vivo [110,123]
	AChE	Altered AChE activity.	In vivo [137,138]
	Monoamine Neurotransmitters	ROS-mediated reduction in monoamine levels.	In vivo [96]
Synaptic Dysplasia	Synapsin and Synaptophysin	Decreased synapsin and synaptophysin expression, dendritic spine loss, and synaptic dysfunction.	In vivo [101,147] In vitro [112]
Autophagy	LC3-II	Increased LC3-II levels: impaired autophagosome turnover.	In vivo [83] In vitro [153]
	p62	Elevated p62 levels: impaired autophagosome degradation.	In vivo [83] In vitro [153]
	Beclin-1	Reduced expression of Beclin-1: impaired pre-autophagosome formation.	In vivo [83] In vitro [153]
Neuroinflammation	TNF-α	Systemic TNF-α elevation and subsequent microglia and astrocyte activation.	In vivo [72,83,123,156,157] In vitro [157]
	IL-1β and IL-6	Dox-induced upregulation of IL-1β and IL-6 via NF-κB pathway.	In vivo [70,83]
	ApoA-I	Dox-induced ApoA-I oxidization and impaired TNF-α regulation.	In vivo [90,158,161] Clinical study [161]
Metabolism	Cholesterol	Disrupted cholesterol metabolism: altered membrane composition and impaired neuronal function.	In vivo [158,159]
	Amino Acid	Altered amino acid levels and dysregulated neurotransmitter synthesis and homeostasis.	In vivo [164]

## References

[1] Ongnok B., Khuanjing T., Chunchai T., Pantiya P., Kerdphoo S., Arunsak B., Nawara W., Jaiwongkam T., Apaijai N., Chattipakorn N. (2021). Donepezil Protects Against Doxorubicin-Induced Chemobrain in Rats via Attenuation of Inflammation and Oxidative Stress Without Interfering With Doxorubicin Efficacy. Neurotherapeutics.

[2] Siegel R.L., Giaquinto A.N., Jemal A. (2024). Cancer Statistics, 2024. CA Cancer J. Clin..

[3] Harbeck N., Saupe S., Jäger E., Schmidt M., Kreienberg R., Müller L., Otremba B.J., Waldenmaier D., Dorn J., Warm M. (2017). A Randomized Phase III Study Evaluating Pegylated Liposomal Doxorubicin versus Capecitabine as First-Line Therapy for Metastatic Breast Cancer: Results of the PELICAN Study. Breast Cancer Res. Treat..

[4] Pisano C., Cecere S.C., Di Napoli M., Cavaliere C., Tambaro R., Facchini G., Scaffa C., Losito S., Pizzolorusso A., Pignata S. (2013). Clinical Trials with Pegylated Liposomal Doxorubicin in the Treatment of Ovarian Cancer. J. Drug Deliv..

[5] Fukuokaya W., Kimura T., Miki J., Kimura S., Watanabe H., Bo F., Okada D., Aikawa K., Ochi A., Suzuki K. (2020). Effectiveness of Intravesical Doxorubicin Immediately Following Resection of Primary Non-Muscle-Invasive Bladder Cancer: A Propensity Score-Matched Analysis. Clin. Genitourin. Cancer.

[6] Al-Gallab M.I., Naddaf L.A., Kanan M.R. (2009). The Management of Non-Invasive Bladder Tumours with Doxorubicin Intravesical Instillation after Transurethral Resection. Sultan Qaboos Univ. Med. J..

[7] Cho H.J., Sim N.S., Shin S.-J., Yun K.-H., Lee Y.H., Jun H.J., Rha S.Y., Kim H.S. (2024). Phase IB/II Trial of Durvalumab plus Doxorubicin Combination in Patients with Advanced Soft-Tissue Sarcoma. J. Clin. Oncol..

[8] Pisters P.W.T., Patel S.R., Prieto V.G., Thall P.F., Lewis V.O., Feig B.W., Hunt K.K., Yasko A.W., Lin P.P., Jacobson M.G. (2004). Phase I Trial of Preoperative Doxorubicin-Based Concurrent Chemoradiation and Surgical Resection for Localized Extremity and Body Wall Soft Tissue Sarcomas. J. Clin. Oncol..

[9] Kantarjian H.M., Walters R.S., Keating M.J., Smith T.L., O’Brien S., Estey E.H., Huh Y.O., Spinolo J., Dicke K., Barlogie B. (1990). Results of the Vincristine, Doxorubicin, and Dexamethasone Regimen in Adults with Standard- and High-Risk Acute Lymphocytic Leukemia. J. Clin. Oncol..

[10] Mattioli R., Ilari A., Colotti B., Mosca L., Fazi F., Colotti G. (2023). Doxorubicin and Other Anthracyclines in Cancers: Activity, Chemoresistance and Its Overcoming. Mol. Asp. Med..

[11] Yang F., Teves S.S., Kemp C.J., Henikoff S. (2014). Doxorubicin, DNA Torsion, and Chromatin Dynamics. Biochim. Biophys. Acta.

[12] Nguyen H.C., Frisbee J.C., Singh K.K. (2024). Different Mechanisms in Doxorubicin-Induced Cardiomyopathy: Impact of BRCA1 and BRCA2 Mutations. Hearts.

[13] Bedard K., Krause K.-H. (2007). The NOX Family of ROS-Generating NADPH Oxidases: Physiology and Pathophysiology. Physiol. Rev..

[14] Roy R., Chun J., Powell S.N. (2011). BRCA1 and BRCA2: Different Roles in a Common Pathway of Genome Protection. Nat. Rev. Cancer.

[15] Incorvaia L., Badalamenti G., Novo G., Gori S., Cortesi L., Brando C., Cinieri S., Curigliano G., Ricciardi G.R., Toss A. (2024). Anthracycline-Related Cardiotoxicity in Patients with Breast Cancer Harboring Mutational Signature of Homologous Recombination Deficiency (HRD). ESMO Open.

[16] Singh K.K., Shukla P.C., Quan A., Al-Omran M., Lovren F., Pan Y., Brezden-Masley C., Ingram A.J., Stanford W.L., Teoh H. (2013). BRCA1 Is a Novel Target to Improve Endothelial Dysfunction and Retard Atherosclerosis. J. Thorac. Cardiovasc. Surg..

[17] Shukla P.C., Singh K.K., Quan A., Al-Omran M., Teoh H., Lovren F., Cao L., Rovira I.I., Pan Y., Brezden-Masley C. (2011). BRCA1 Is an Essential Regulator of Heart Function and Survival Following Myocardial Infarction. Nat. Commun..

[18] Singh K.K., Shukla P.C., Quan A., Desjardins J.-F., Lovren F., Pan Y., Garg V., Gosal S., Garg A., Szmitko P.E. (2012). BRCA2 Protein Deficiency Exaggerates Doxorubicin-Induced Cardiomyocyte Apoptosis and Cardiac Failure. J. Biol. Chem..

[19] Bhinge K.N., Gupta V., Hosain S.B., Satyanarayanajois S.D., Meyer S.A., Blaylock B., Zhang Q.-J., Liu Y.-Y. (2012). The Opposite Effects of Doxorubicin on Bone Marrow Stem Cells versus Breast Cancer Stem Cells Depend on Glucosylceramide Synthase. Int. J. Biochem. Cell Biol..

[20] Rawat P.S., Jaiswal A., Khurana A., Bhatti J.S., Navik U. (2021). Doxorubicin-Induced Cardiotoxicity: An Update on the Molecular Mechanism and Novel Therapeutic Strategies for Effective Management. Biomed. Pharmacother..

[21] Du J., Zhang A., Li J., Liu X., Wu S., Wang B., Wang Y., Jia H. (2021). Doxorubicin-Induced Cognitive Impairment: The Mechanistic Insights. Front. Oncol..

[22] Ongnok B., Chattipakorn N., Chattipakorn S.C. (2020). Doxorubicin and Cisplatin Induced Cognitive Impairment: The Possible Mechanisms and Interventions. Exp. Neurol..

[23] Berndt U., Leplow B., Schoenfeld R., Lantzsch T., Grosse R., Thomssen C. (2016). Memory and Spatial Cognition in Breast Cancer Patients Undergoing Adjuvant Endocrine Therapy. Breast Care.

[24] Barzilai A., Biton S., Shiloh Y. (2008). The Role of the DNA Damage Response in Neuronal Development, Organization and Maintenance. DNA Repair.

[25] Bigotte L., Arvidson B., Olsson Y. (1982). Cytofluorescence Localization of Adriamycin in the Nervous System. I. Distribution of the Drug in the Central Nervous System of Normal Adult Mice after Intravenous Injection. Acta Neuropathol..

[26] Sardi I., la Marca G., Cardellicchio S., Giunti L., Malvagia S., Genitori L., Massimino M., de Martino M., Giovannini M.G. (2013). Pharmacological Modulation of Blood-Brain Barrier Increases Permeability of Doxorubicin into the Rat Brain. Am. J. Cancer Res..

[27] Zhao Y.L., Du J., Kanazawa H., Sugawara A., Takagi K., Kitaichi K., Tatsumi Y., Takagi K., Hasegawa T. (2002). Effect of Endotoxin on Doxorubicin Transport across Blood-Brain Barrier and P-Glycoprotein Function in Mice. Eur. J. Pharmacol..

[28] Alsikhan R.S., Aldubayan M.A., Almami I.S., Alhowail A.H. (2023). Protective Effect of Galantamine against Doxorubicin-Induced Neurotoxicity. Brain Sci..

[29] Mohamed R.H., Karam R.A., Amer M.G. (2011). Epicatechin Attenuates Doxorubicin-Induced Brain Toxicity: Critical Role of TNF-α, iNOS and NF-κB. Brain Res. Bull.

[30] Obermeier B., Daneman R., Ransohoff R.M. (2013). Development, Maintenance and Disruption of the Blood-Brain Barrier. Nat. Med..

[31] Kadry H., Noorani B., Cucullo L. (2020). A Blood-Brain Barrier Overview on Structure, Function, Impairment, and Biomarkers of Integrity. Fluids Barriers CNS.

[32] Sardi I., Fantappiè O., la Marca G., Giovannini M.G., Iorio A.L., da Ros M., Malvagia S., Cardellicchio S., Giunti L., de Martino M. (2014). Delivery of Doxorubicin across the Blood-Brain Barrier by Ondansetron Pretreatment: A Study in Vitro and in Vivo. Cancer Lett..

[33] Wallez Y., Huber P. (2008). Endothelial Adherens and Tight Junctions in Vascular Homeostasis, Inflammation and Angiogenesis. Biochim. Biophys. Acta.

[34] Wilkinson E.L., Sidaway J.E., Cross M.J. (2016). Cardiotoxic Drugs Herceptin and Doxorubicin Inhibit Cardiac Microvascular Endothelial Cell Barrier Formation Resulting in Increased Drug Permeability. Biol. Open.

[35] Luu A.Z., Chowdhury B., Al-Omran M., Teoh H., Hess D.A., Verma S. (2018). Role of Endothelium in Doxorubicin-Induced Cardiomyopathy. JACC Basic Transl. Sci..

[36] Licht T., Sasson E., Bell B., Grunewald M., Kumar S., Kreisel T., Ben-Zvi A., Keshet E. (2020). Hippocampal Neural Stem Cells Facilitate Access from Circulation via Apical Cytoplasmic Processes. Elife.

[37] Sung H., Ferlay J., Siegel R.L., Laversanne M., Soerjomataram I., Jemal A., Bray F. (2021). Global Cancer Statistics 2020: GLOBOCAN Estimates of Incidence and Mortality Worldwide for 36 Cancers in 185 Countries. CA Cancer J. Clin..

[38] Shen L., Zhang S., Wang K., Wang X. (2021). Familial Breast Cancer: Disease Related Gene Mutations and Screening Strategies for Chinese Population. Front. Oncol..

[39] Kotsopoulos J. (2018). BRCA Mutations and Breast Cancer Prevention. Cancers.

[40] Marmorstein L.Y., Ouchi T., Aaronson S.A. (1998). The BRCA2 Gene Product Functionally Interacts with P53 and RAD51. Proc. Natl. Acad. Sci. USA.

[41] Griendling K.K., FitzGerald G.A. (2003). Oxidative Stress and Cardiovascular Injury: Part II: Animal and Human Studies. Circulation.

[42] Robert G., Wagner J.R. (2020). ROS-Induced DNA Damage as an Underlying Cause of Aging. Adv. Geriatr. Med. Res..

[43] O’Driscoll M. (2012). Diseases Associated with Defective Responses to DNA Damage. Cold Spring Harb. Perspect. Biol..

[44] Loughery J., Cox M., Smith L.M., Meek D.W. (2014). Critical Role for P53-Serine 15 Phosphorylation in Stimulating Transactivation at P53-Responsive Promoters. Nucleic Acids Res..

[45] Meyer T., Jahn N., Lindner S., Röhner L., Dolnik A., Weber D., Scheffold A., Köpff S., Paschka P., Gaidzik V.I. (2020). Functional Characterization of BRCC3 Mutations in Acute Myeloid Leukemia with t(8;21)(Q22;Q22.1). Leukemia.

[46] Mao Z., Bozzella M., Seluanov A., Gorbunova V. (2008). DNA Repair by Nonhomologous End Joining and Homologous Recombination during Cell Cycle in Human Cells. Cell Cycle.

[47] Lei T., Du S., Peng Z., Chen L. (2022). Multifaceted Regulation and Functions of 53BP1 in NHEJ-mediated DSB Repair (Review). Int. J. Mol. Med..

[48] Rappold I., Iwabuchi K., Date T., Chen J. (2001). Tumor Suppressor P53 Binding Protein 1 (53BP1) Is Involved in DNA Damage-Signaling Pathways. J. Cell Biol..

[49] Zhao F., Kim W., Kloeber J.A., Lou Z. (2020). DNA End Resection and Its Role in DNA Replication and DSB Repair Choice in Mammalian Cells. Exp. Mol. Med..

[50] Bunting S.F., Callén E., Wong N., Chen H.-T., Polato F., Gunn A., Bothmer A., Feldhahn N., Fernandez-Capetillo O., Cao L. (2010). 53BP1 Inhibits Homologous Recombination in Brca1-Deficient Cells by Blocking Resection of DNA Breaks. Cell.

[51] Krasner D.S., Daley J.M., Sung P., Niu H. (2015). Interplay between Ku and Replication Protein A in the Restriction of Exo1-Mediated DNA Break End Resection. J. Biol. Chem..

[52] Achanta G., Pelicano H., Feng L., Plunkett W., Huang P. (2001). Interaction of P53 and DNA-PK in Response to Nucleoside Analogues: Potential Role as a Sensor Complex for DNA Damage. Cancer Res..

[53] MacLachlan T.K., Takimoto R., El-Deiry W.S. (2002). BRCA1 Directs a Selective P53-Dependent Transcriptional Response towards Growth Arrest and DNA Repair Targets. Mol. Cell. Biol..

[54] Bae I., Fan S., Meng Q., Rih J.K., Kim H.J., Kang H.J., Xu J., Goldberg I.D., Jaiswal A.K., Rosen E.M. (2004). BRCA1 Induces Antioxidant Gene Expression and Resistance to Oxidative Stress. Cancer Res..

[55] Xu P., Liu Q., Xie Y., Shi X., Li Y., Peng M., Guo H., Sun R., Li J., Hong Y. (2018). Breast Cancer Susceptibility Protein 1 (BRCA1) Rescues Neurons from Cerebral Ischemia/Reperfusion Injury through NRF2-Mediated Antioxidant Pathway. Redox Biol..

[56] Gorrini C., Baniasadi P.S., Harris I.S., Silvester J., Inoue S., Snow B., Joshi P.A., Wakeham A., Molyneux S.D., Martin B. (2013). BRCA1 Interacts with Nrf2 to Regulate Antioxidant Signaling and Cell Survival. J. Exp. Med..

[57] Wang C., Gao P., Xu J., Liu S., Tian W., Liu J., Zhou L. (2022). Natural Phytochemicals Prevent Side Effects in BRCA-Mutated Ovarian Cancer and PARP Inhibitor Treatment. Front. Pharmacol..

[58] Ma J., Cai H., Wu T., Sobhian B., Huo Y., Alcivar A., Mehta M., Cheung K.L., Ganesan S., Kong A.-N.T. (2012). PALB2 Interacts with KEAP1 to Promote NRF2 Nuclear Accumulation and Function. Mol. Cell Biol..

[59] Deng C.-X. (2006). BRCA1: Cell Cycle Checkpoint, Genetic Instability, DNA Damage Response and Cancer Evolution. Nucleic Acids Res..

[60] Aprelikova O.N., Fang B.S., Meissner E.G., Cotter S., Campbell M., Kuthiala A., Bessho M., Jensen R.A., Liu E.T. (1999). BRCA1-Associated Growth Arrest Is RB-Dependent. Proc. Natl. Acad. Sci. USA.

[61] Ross C.A., Truant R. (2017). DNA Repair: A Unifying Mechanism in Neurodegeneration. Nature.

[62] Naumann M., Pal A., Goswami A., Lojewski X., Japtok J., Vehlow A., Naujock M., Günther R., Jin M., Stanslowsky N. (2018). Impaired DNA Damage Response Signaling by FUS-NLS Mutations Leads to Neurodegeneration and FUS Aggregate Formation. Nat. Commun..

[63] Marques L., Johnson A.A., Stolzing A. (2020). Doxorubicin Generates Senescent Microglia That Exhibit Altered Proteomes, Higher Levels of Cytokine Secretion, and a Decreased Ability to Internalize Amyloid β. Exp. Cell Res..

[64] Lavigne M.C., Malech H.L., Holland S.M., Leto T.L. (2001). Genetic Requirement of P47phox for Superoxide Production by Murine Microglia. FASEB J..

[65] Polidori M.C., Griffiths H.R., Mariani E., Mecocci P. (2007). Hallmarks of Protein Oxidative Damage in Neurodegenerative Diseases: Focus on Alzheimer’s Disease. Amino Acids.

[66] Barnham K.J., Masters C.L., Bush A.I. (2004). Neurodegenerative Diseases and Oxidative Stress. Nat. Rev. Drug Discov..

[67] Fukai T., Ushio-Fukai M. (2011). Superoxide Dismutases: Role in Redox Signaling, Vascular Function, and Diseases. Antioxid Redox Signal..

[68] Lopes M.A., Meisel A., Carvalho F.D., de Lourdes Bastos M. (2011). Neuronal Nitric Oxide Synthase Is a Key Factor in Doxorubicin-Induced Toxicity to Rat-Isolated Cortical Neurons. Neurotox Res..

[69] Ren X., Keeney J.T.R., Miriyala S., Noel T., Powell D.K., Chaiswing L., Bondada S., St. Clair D.K., Butterfield D.A. (2019). The Triangle of Death of Neurons: Oxidative Damage, Mitochondrial Dysfunction, and Loss of Choline-Containing Biomolecules in Brains of Mice Treated with Doxorubicin. Advanced Insights into Mechanisms of Chemotherapy Induced Cognitive Impairment (“Chemobrain”) Involving TNF-α. Free. Radic. Biol. Med..

[70] Wu Y.-Q., Dang R.-L., Tang M.-M., Cai H.-L., Li H.-D., Liao D.-H., He X., Cao L.-J., Xue Y., Jiang P. (2016). Long Chain Omega-3 Polyunsaturated Fatty Acid Supplementation Alleviates Doxorubicin-Induced Depressive-Like Behaviors and Neurotoxicity in Rats: Involvement of Oxidative Stress and Neuroinflammation. Nutrients.

[71] Keeney J.T.R., Förster S., Sultana R., Brewer L.D., Latimer C.S., Cai J., Klein J.B., Porter N.M., Butterfield D.A. (2013). Dietary Vitamin D Deficiency in Rats from Middle to Old Age Leads to Elevated Tyrosine Nitration and Proteomics Changes in Levels of Key Proteins in Brain: Implications for Low Vitamin D-Dependent Age-Related Cognitive Decline. Free Radic. Biol. Med..

[72] Liao D., Xiang D., Dang R., Xu P., Wang J., Han W., Fu Y., Yao D., Cao L., Jiang P. (2018). Neuroprotective Effects of dl-3-n-Butylphthalide against Doxorubicin-Induced Neuroinflammation, Oxidative Stress, Endoplasmic Reticulum Stress, and Behavioral Changes. Oxidative Med. Cell. Longev..

[73] Conrad M., Friedmann Angeli J.P. (2015). Glutathione Peroxidase 4 (Gpx4) and Ferroptosis: What’s so Special about It?. Mol. Cell Oncol..

[74] Imosemi I.O., Owumi S.E., Arunsi U.O. (2022). Biochemical and Histological Alterations of Doxorubicin-Induced Neurotoxicity in Rats: Protective Role of Luteolin. J. Biochem. Mol. Toxicol..

[75] Da-Silva O.F., Adelowo A.R., Babalola A.A., Ikeji C.N., Owoeye O., Rocha J.B.T., Adedara I.A., Farombi E.O. (2024). Diphenyl Diselenide Through Reduction of Inflammation, Oxidative Injury and Caspase-3 Activation Abates Doxorubicin-Induced Neurotoxicity in Rats. Neurochem. Res..

[76] Zhang H.-J., Fu Y., Zhang H., Lai Z.-Q., Dong Y.-F. (2024). Sophocarpine Alleviates Doxorubicin-Induced Heart Injury by Suppressing Oxidative Stress and Apoptosis. Sci. Rep..

[77] Patra R.C., Swarup D., Dwivedi S.K. (2001). Antioxidant Effects of Alpha Tocopherol, Ascorbic Acid and L-Methionine on Lead Induced Oxidative Stress to the Liver, Kidney and Brain in Rats. Toxicology.

[78] Fransen M., Nordgren M., Wang B., Apanasets O. (2012). Role of Peroxisomes in ROS/RNS-Metabolism: Implications for Human Disease. Biochim. Biophys. Acta.

[79] Ayala A., Muñoz M.F., Argüelles S. (2014). Lipid Peroxidation: Production, Metabolism, and Signaling Mechanisms of Malondialdehyde and 4-Hydroxy-2-Nonenal. Oxid. Med. Cell Longev..

[80] Keeney J.T.R., Ren X., Warrier G., Noel T., Powell D.K., Brelsfoard J.M., Sultana R., Saatman K.E., St. Clair D.K., Butterfield D.A. (2018). Doxorubicin-Induced Elevated Oxidative Stress and Neurochemical Alterations in Brain and Cognitive Decline: Protection by MESNA and Insights into Mechanisms of Chemotherapy-Induced Cognitive Impairment (“Chemobrain”). Oncotarget.

[81] Zhou X., Xu P., Dang R., Guo Y., Li G., Qiao Y., Xie R., Liu Y., Jiang P. (2018). The Involvement of Autophagic Flux in the Development and Recovery of Doxorubicin-Induced Neurotoxicity. Free Radic. Biol. Med..

[82] Moruno-Manchon J.F., Uzor N.-E., Kesler S.R., Wefel J.S., Townley D.M., Nagaraja A.S., Pradeep S., Mangala L.S., Sood A.K., Tsvetkov A.S. (2018). Peroxisomes Contribute to Oxidative Stress in Neurons during Doxorubicin-Based Chemotherapy. Mol. Cell Neurosci..

[83] Tangpong J., Cole M.P., Sultana R., Joshi G., Estus S., Vore M., St Clair W., Ratanachaiyavong S., St Clair D.K., Butterfield D.A. (2006). Adriamycin-Induced, TNF-Alpha-Mediated Central Nervous System Toxicity. Neurobiol. Dis..

[84] Wallace K.B., Sardão V.A., Oliveira P.J. (2020). Mitochondrial Determinants of Doxorubicin-Induced Cardiomyopathy. Circ. Res..

[85] Alhowail A.H., Bloemer J., Majrashi M., Pinky P.D., Bhattacharya S., Yongli Z., Bhattacharya D., Eggert M., Woodie L., Buabeid M.A. (2019). Doxorubicin-Induced Neurotoxicity Is Associated with Acute Alterations in Synaptic Plasticity, Apoptosis, and Lipid Peroxidation. Toxicol. Mech. Methods.

[86] Dias-Carvalho A., Ferreira M., Reis-Mendes A., Ferreira R., de Lourdes Bastos M., Fernandes E., Sá S.I., Capela J.P., Carvalho F., Costa V.M. (2024). Doxorubicin-Induced Neurotoxicity Differently Affects the Hippocampal Formation Subregions in Adult Mice. Heliyon.

[87] Cardoso S., Santos R.X., Carvalho C., Correia S., Pereira G.C., Pereira S.S., Oliveira P.J., Santos M.S., Proença T., Moreira P.I. (2008). Doxorubicin Increases the Susceptibility of Brain Mitochondria to Ca(2+)-Induced Permeability Transition and Oxidative Damage. Free Radic. Biol. Med..

[88] Maynard S., Fang E.F., Scheibye-Knudsen M., Croteau D.L., Bohr V.A. (2015). DNA Damage, DNA Repair, Aging, and Neurodegeneration. Cold Spring Harb. Perspect. Med..

[89] Holley A.K., Bakthavatchalu V., Velez-Roman J.M., St Clair D.K. (2011). Manganese Superoxide Dismutase: Guardian of the Powerhouse. Int. J. Mol. Sci..

[90] Tangpong J., Cole M.P., Sultana R., Estus S., Vore M., St Clair W., Ratanachaiyavong S., St Clair D.K., Butterfield D.A. (2007). Adriamycin-Mediated Nitration of Manganese Superoxide Dismutase in the Central Nervous System: Insight into the Mechanism of Chemobrain. J. Neurochem..

[91] Pugazhendhi A., Edison T.N.J.I., Velmurugan B.K., Jacob J.A., Karuppusamy I. (2018). Toxicity of Doxorubicin (Dox) to Different Experimental Organ Systems. Life Sci..

[92] Carozzi V.A., Canta A., Chiorazzi A. (2015). Chemotherapy-Induced Peripheral Neuropathy: What Do We Know about Mechanisms?. Neurosci. Lett..

[93] Melli G., Taiana M., Camozzi F., Triolo D., Podini P., Quattrini A., Taroni F., Lauria G. (2008). Alpha-Lipoic Acid Prevents Mitochondrial Damage and Neurotoxicity in Experimental Chemotherapy Neuropathy. Exp. Neurol..

[94] Pellacani C., Eleftheriou G. (2020). Neurotoxicity of Antineoplastic Drugs: Mechanisms, Susceptibility, and Neuroprotective Strategies. Adv. Med. Sci..

[95] Merzoug S., Toumi M.L., Tahraoui A. (2014). Quercetin Mitigates Adriamycin-Induced Anxiety- and Depression-like Behaviors, Immune Dysfunction, and Brain Oxidative Stress in Rats. Naunyn Schmiedebergs Arch. Pharmacol..

[96] Kwatra M., Jangra A., Mishra M., Sharma Y., Ahmed S., Ghosh P., Kumar V., Vohora D., Khanam R. (2016). Naringin and Sertraline Ameliorate Doxorubicin-Induced Behavioral Deficits Through Modulation of Serotonin Level and Mitochondrial Complexes Protection Pathway in Rat Hippocampus. Neurochem. Res..

[97] Okudan N., Belviranlı M., Sezer T. (2022). Potential Protective Effect of Coenzyme Q10 on Doxorubicin-Induced Neurotoxicity and Behavioral Disturbances in Rats. Neurochem. Res..

[98] Ebrahim N.A., Elnagar M.R., El-Gamal R., Habotta O.A., Albadawi E.A., Albadrani M., Bahashwan A.S., Hassan H.M. (2024). Melatonin Mitigates Doxorubicin Induced Chemo Brain in a Rat Model in a NRF2/P53-SIRT1 Dependent Pathway. Heliyon.

[99] Fu Z., Guo J., Jing L., Li R., Zhang T., Peng S. (2010). Enhanced Toxicity and ROS Generation by Doxorubicin in Primary Cultures of Cardiomyocytes from Neonatal Metallothionein-I/II Null Mice. Toxicol. Vitr..

[100] Tangpong J., Miriyala S., Noel T., Sinthupibulyakit C., Jungsuwadee P., St Clair D.K. (2011). Doxorubicin-Induced Central Nervous System Toxicity and Protection by Xanthone Derivative of Garcinia Mangostana. Neuroscience.

[101] El-Shetry E.S., Ibrahim I.A., Kamel A.M., Abdelwahab O.A. (2024). Quercetin Mitigates Doxorubicin-Induced Neurodegenerative Changes in the Cerebral Cortex and Hippocampus of Rats; Insights to DNA Damage, Inflammation, Synaptic Plasticity. Tissue Cell.

[102] Ibrahim Fouad G., Ahmed K.A. (2021). Neuroprotective Potential of Berberine Against Doxorubicin-Induced Toxicity in Rat’s Brain. Neurochem. Res..

[103] Chao X., Zhou J., Chen T., Liu W., Dong W., Qu Y., Jiang X., Ji X., Zhen H., Fei Z. (2010). Neuroprotective Effect of Osthole against Acute Ischemic Stroke on Middle Cerebral Ischemia Occlusion in Rats. Brain Res..

[104] Ogawa H., Sasai N., Kamisako T., Baba K. (2007). Effects of Osthol on Blood Pressure and Lipid Metabolism in Stroke-Prone Spontaneously Hypertensive Rats. J. Ethnopharmacol..

[105] Shokoohinia Y., Hosseinzadeh L., Moieni-Arya M., Mostafaie A., Mohammadi-Motlagh H.-R. (2014). Osthole Attenuates Doxorubicin-Induced Apoptosis in PC12 Cells through Inhibition of Mitochondrial Dysfunction and ROS Production. Biomed. Res. Int..

[106] Kohman R.A., Rhodes J.S. (2013). Neurogenesis, Inflammation and Behavior. Brain Behav. Immun..

[107] Kempermann G., Song H., Gage F.H. (2015). Neurogenesis in the Adult Hippocampus. Cold Spring Harb. Perspect. Biol..

[108] Christie L.-A., Acharya M.M., Parihar V.K., Nguyen A., Martirosian V., Limoli C.L. (2012). Impaired Cognitive Function and Hippocampal Neurogenesis Following Cancer Chemotherapy. Clin. Cancer Res..

[109] Sekeres M.J., Bradley-Garcia M., Martinez-Canabal A., Winocur G. (2021). Chemotherapy-Induced Cognitive Impairment and Hippocampal Neurogenesis: A Review of Physiological Mechanisms and Interventions. Int. J. Mol. Sci..

[110] Alhowail A.H., Pinky P.D., Eggert M., Bloemer J., Woodie L.N., Buabeid M.A., Bhattacharya S., Jasper S.L., Bhattacharya D., Dhanasekaran M. (2021). Doxorubicin Induces Dysregulation of AMPA Receptor and Impairs Hippocampal Synaptic Plasticity Leading to Learning and Memory Deficits. Heliyon.

[111] Kitamura Y., Hattori S., Yoneda S., Watanabe S., Kanemoto E., Sugimoto M., Kawai T., Machida A., Kanzaki H., Miyazaki I. (2015). Doxorubicin and Cyclophosphamide Treatment Produces Anxiety-like Behavior and Spatial Cognition Impairment in Rats: Possible Involvement of Hippocampal Neurogenesis via Brain-Derived Neurotrophic Factor and Cyclin D1 Regulation. Behav. Brain Res..

[112] Manchon J.F.M., Dabaghian Y., Uzor N.-E., Kesler S.R., Wefel J.S., Tsvetkov A.S. (2016). Levetiracetam Mitigates Doxorubicin-Induced DNA and Synaptic Damage in Neurons. Sci. Rep..

[113] Kitamura Y., Kanemoto E., Sugimoto M., Machida A., Nakamura Y., Naito N., Kanzaki H., Miyazaki I., Asanuma M., Sendo T. (2017). Influence of Nicotine on Doxorubicin and Cyclophosphamide Combination Treatment-Induced Spatial Cognitive Impairment and Anxiety-like Behavior in Rats. Naunyn Schmiedebergs Arch. Pharmacol..

[114] Ramalingayya G.V., Cheruku S.P., Nayak P.G., Kishore A., Shenoy R., Rao C.M., Krishnadas N. (2017). Rutin Protects against Neuronal Damage in Vitro and Ameliorates Doxorubicin-Induced Memory Deficits in Vivo in Wistar Rats. Drug Des. Devel. Ther..

[115] Gao Y., Dong J., Chen M., Wang T., Yang Z., He K., Li Y., Wang K., Jiang J., Zhang S. (2022). Protective Effect of Low-Dose Radiation on Doxorubicin-Induced Brain Injury in Mice. Arch. Biochem. Biophys..

[116] Bigotte L., Olsson Y. (1983). Toxic Effects of Adriamycin on the Central Nervous System. Ultrastructural Changes in Some Circumventricular Organs of the Mouse after Intravenous Administration of the Drug. Acta Neuropathol..

[117] Matusova Z., Hol E.M., Pekny M., Kubista M., Valihrach L. (2023). Reactive Astrogliosis in the Era of Single-Cell Transcriptomics. Front. Cell Neurosci..

[118] Deuker L., Doeller C.F., Fell J., Axmacher N. (2014). Human Neuroimaging Studies on the Hippocampal CA3 Region—Integrating Evidence for Pattern Separation and Completion. Front. Cell Neurosci..

[119] van Praag H., Christie B.R., Sejnowski T.J., Gage F.H. (1999). Running Enhances Neurogenesis, Learning, and Long-Term Potentiation in Mice. Proc. Natl. Acad. Sci. USA.

[120] van Praag H., Kempermann G., Gage F.H. (1999). Running Increases Cell Proliferation and Neurogenesis in the Adult Mouse Dentate Gyrus. Nat. Neurosci..

[121] Mohamad H.E., Abo-Elmatty D.M., Wahba N.S., Shaheen M.A., Sakr R.T., Wahba A.S. (2022). Infliximab and/or MESNA Alleviate Doxorubicin-Induced Alzheimer’s Disease-like Pathology in Rats: A New Insight into TNF-α/Wnt/β-Catenin Signaling Pathway. Life Sci..

[122] Yang M., Kim J.-S., Kim J., Kim S.-H., Kim J.-C., Kim J., Wang H., Shin T., Moon C. (2011). Neurotoxicity of Methotrexate to Hippocampal Cells in Vivo and in Vitro. Biochem. Pharmacol..

[123] Alhowail A.H., Aldubayan M.A. (2023). Doxorubicin Impairs Cognitive Function by Upregulating AMPAR and NMDAR Subunit Expression and Increasing Neuroinflammation, Oxidative Stress, and Apoptosis in the Brain. Front. Pharmacol..

[124] Pal M.M. (2021). Glutamate: The Master Neurotransmitter and Its Implications in Chronic Stress and Mood Disorders. Front. Hum. Neurosci..

[125] Endlicher R., Drahota Z., Štefková K., Červinková Z., Kučera O. (2023). The Mitochondrial Permeability Transition Pore-Current Knowledge of Its Structure, Function, and Regulation, and Optimized Methods for Evaluating Its Functional State. Cells.

[126] Matuz-Mares D., González-Andrade M., Araiza-Villanueva M.G., Vilchis-Landeros M.M., Vázquez-Meza H. (2022). Mitochondrial Calcium: Effects of Its Imbalance in Disease. Antioxidants.

[127] Uryash A., Flores V., Adams J.A., Allen P.D., Lopez J.R. (2020). Memory and Learning Deficits Are Associated With Ca2+ Dyshomeostasis in Normal Aging. Front. Aging Neurosci..

[128] Belov Kirdajova D., Kriska J., Tureckova J., Anderova M. (2020). Ischemia-Triggered Glutamate Excitotoxicity From the Perspective of Glial Cells. Front. Cell Neurosci..

[129] Thomas T.C., Beitchman J.A., Pomerleau F., Noel T., Jungsuwadee P., Butterfield D.A., Clair D.K.S., Vore M., Gerhardt G.A. (2017). Acute Treatment with Doxorubicin Affects Glutamate Neurotransmission in the Mouse Frontal Cortex and Hippocampus. Brain Res..

[130] Mahmoud S., Gharagozloo M., Simard C., Gris D. (2019). Astrocytes Maintain Glutamate Homeostasis in the CNS by Controlling the Balance between Glutamate Uptake and Release. Cells.

[131] Todd A.C., Hardingham G.E. (2020). The Regulation of Astrocytic Glutamate Transporters in Health and Neurodegenerative Diseases. Int. J. Mol. Sci..

[132] Su Z., Leszczyniecka M., Kang D., Sarkar D., Chao W., Volsky D.J., Fisher P.B. (2003). Insights into Glutamate Transport Regulation in Human Astrocytes: Cloning of the Promoter for Excitatory Amino Acid Transporter 2 (EAAT2). Proc. Natl. Acad. Sci. USA.

[133] Cao L., Li L., Zuo Z. (2012). N-Acetylcysteine Reverses Existing Cognitive Impairment and Increased Oxidative Stress in Glutamate Transporter Type 3 Deficient Mice. Neuroscience.

[134] Ezeriņa D., Takano Y., Hanaoka K., Urano Y., Dick T.P. (2018). N-Acetyl Cysteine Functions as a Fast-Acting Antioxidant by Triggering Intracellular H2S and Sulfane Sulfur Production. Cell Chem. Biol..

[135] Takahashi K., Foster J.B., Lin C.-L.G. (2015). Glutamate Transporter EAAT2: Regulation, Function, and Potential as a Therapeutic Target for Neurological and Psychiatric Disease. Cell Mol. Life Sci..

[136] Picciotto M.R., Higley M.J., Mineur Y.S. (2012). Acetylcholine as a Neuromodulator: Cholinergic Signaling Shapes Nervous System Function and Behavior. Neuron.

[137] El-Agamy S.E., Abdel-Aziz A.K., Wahdan S., Esmat A., Azab S.S. (2018). Astaxanthin Ameliorates Doxorubicin-Induced Cognitive Impairment (Chemobrain) in Experimental Rat Model: Impact on Oxidative, Inflammatory, and Apoptotic Machineries. Mol. Neurobiol..

[138] Khadrawy Y.A., Hosny E.N., Mohammed H.S. (2021). Protective Effect of Nanocurcumin against Neurotoxicity Induced by Doxorubicin in Rat’s Brain. Neurotoxicology.

[139] Garcia-Ratés S., Greenfield S. (2017). Cancer and Neurodegeneration: Two Sides, Same Coin?. Oncotarget.

[140] Onganer P.U., Djamgoz M.B.A., Whyte K., Greenfield S.A. (2006). An Acetylcholinesterase-Derived Peptide Inhibits Endocytic Membrane Activity in a Human Metastatic Breast Cancer Cell Line. Biochim. Biophys. Acta.

[141] Greenfield S. (2013). Discovering and Targeting the Basic Mechanism of Neurodegeneration: The Role of Peptides from the C-Terminus of Acetylcholinesterase: Non-Hydrolytic Effects of Ache: The Actions of Peptides Derived from the C-Terminal and Their Relevance to Neurodegeneration. Chem. Biol. Interact..

[142] Lim I., Joung H.-Y., Yu A.R., Shim I., Kim J.S. (2016). PET Evidence of the Effect of Donepezil on Cognitive Performance in an Animal Model of Chemobrain. Biomed. Res. Int..

[143] Bortolato M., Chen K., Shih J.C. (2008). Monoamine Oxidase Inactivation: From Pathophysiology to Therapeutics. Adv. Drug Deliv. Rev..

[144] Petrovic M., Simillion C., Kruzliak P., Sabo J., Heller M. (2015). Doxorubicin Affects Expression of Proteins of Neuronal Pathways in MCF-7 Breast Cancer Cells. Cancer Genom. Proteom..

[145] Ugalde-Triviño L., Díaz-Guerra M. (2021). PSD-95: An Effective Target for Stroke Therapy Using Neuroprotective Peptides. Int. J. Mol. Sci..

[146] Vallejo D., Codocedo J.F., Inestrosa N.C. (2017). Posttranslational Modifications Regulate the Postsynaptic Localization of PSD-95. Mol. Neurobiol..

[147] Ansari M.A., Roberts K.N., Scheff S.W. (2008). Oxidative Stress and Modification of Synaptic Proteins in Hippocampus after Traumatic Brain Injury. Free Radic. Biol. Med..

[148] Kandlur A., Satyamoorthy K., Gangadharan G. (2020). Oxidative Stress in Cognitive and Epigenetic Aging: A Retrospective Glance. Front. Mol. Neurosci..

[149] Bu S., Joseph J.J., Nguyen H.C., Ehsan M., Rasheed B., Singh A., Qadura M., Frisbee J.C., Singh K.K. (2023). MicroRNA miR-378-3p Is a Novel Regulator of Endothelial Autophagy and Function. J. Mol. Cell Cardiol. Plus.

[150] Singh A., Ravendranathan N., Frisbee J.C., Singh K.K. (2024). Complex Interplay between DNA Damage and Autophagy in Disease and Therapy. Biomolecules.

[151] Van Limbergen J., Stevens C., Nimmo E.R., Wilson D.C., Satsangi J. (2009). Autophagy: From Basic Science to Clinical Application. Mucosal Immunol..

[152] Myerowitz R., Puertollano R., Raben N. (2021). Impaired Autophagy: The Collateral Damage of Lysosomal Storage Disorders. EBioMedicine.

[153] Moruno-Manchon J.F., Uzor N.-E., Kesler S.R., Wefel J.S., Townley D.M., Nagaraja A.S., Pradeep S., Mangala L.S., Sood A.K., Tsvetkov A.S. (2016). TFEB Ameliorates the Impairment of the Autophagy-Lysosome Pathway in Neurons Induced by Doxorubicin. Aging.

[154] Liao D., Shangguan D., Wu Y., Chen Y., Liu N., Tang J., Yao D., Shi Y. (2023). Curcumin Protects against Doxorubicin Induced Oxidative Stress by Regulating the Keap1-Nrf2-ARE and Autophagy Signaling Pathways. Psychopharmacology.

[155] Song W., Wang F., Lotfi P., Sardiello M., Segatori L. (2014). 2-Hydroxypropyl-β-Cyclodextrin Promotes Transcription Factor EB-Mediated Activation of Autophagy: Implications for Therapy. J. Biol. Chem..

[156] Habbas S., Santello M., Becker D., Stubbe H., Zappia G., Liaudet N., Klaus F.R., Kollias G., Fontana A., Pryce C.R. (2015). Neuroinflammatory TNFα Impairs Memory via Astrocyte Signaling. Cell.

[157] Brás J.P., Bravo J., Freitas J., Barbosa M.A., Santos S.G., Summavielle T., Almeida M.I. (2020). TNF-Alpha-Induced Microglia Activation Requires miR-342: Impact on NF-kB Signaling and Neurotoxicity. Cell Death Dis..

[158] Keeney J.T.R., Miriyala S., Noel T., Moscow J.A., St Clair D.K., Butterfield D.A. (2015). Superoxide Induces Protein Oxidation in Plasma and TNF-α Elevation in Macrophage Culture: Insights into Mechanisms of Neurotoxicity Following Doxorubicin Chemotherapy. Cancer Lett..

[159] Geng C., Cui C., Wang C., Lu S., Zhang M., Chen D., Jiang P. (2021). Systematic Evaluations of Doxorubicin-Induced Toxicity in Rats Based on Metabolomics. ACS Omega.

[160] Lal R., Dharavath R.N., Chopra K. (2023). Alpha-Lipoic Acid Ameliorates Doxorubicin-Induced Cognitive Impairments by Modulating Neuroinflammation and Oxidative Stress via NRF-2/HO-1 Signaling Pathway in the Rat Hippocampus. Neurochem. Res..

[161] Aluise C.D., Miriyala S., Noel T., Sultana R., Jungsuwadee P., Taylor T.J., Cai J., Pierce W.M., Vore M., Moscow J.A. (2011). 2-Mercaptoethane Sulfonate Prevents Doxorubicin-Induced Plasma Protein Oxidation and TNF-α Release: Implications for the Reactive Oxygen Species-Mediated Mechanisms of Chemobrain. Free Radic. Biol. Med..

[162] Shao B., Tang C., Heinecke J.W., Oram J.F. (2010). Oxidation of Apolipoprotein A-I by Myeloperoxidase Impairs the Initial Interactions with ABCA1 Required for Signaling and Cholesterol Export. J. Lipid Res..

[163] Yu X., Guo L., Deng X., Yang F., Tian Y., Liu P., Xu F., Zhang Z., Huang Y. (2021). Attenuation of Doxorubicin-Induced Oxidative Damage in Rat Brain by Regulating Amino Acid Homeostasis with Astragali Radix. Amino Acids.

[164] Liu P., Guo L., Yu X., Liu P., Yu Y., Kong X., Yu X., Zephania H.M., Liu P., Huang Y. (2023). Identification of Region-Specific Amino Acid Signatures for Doxorubicin-Induced Chemo Brain. Amino Acids.

[165] Guerriero R.M., Giza C.C., Rotenberg A. (2015). Glutamate and GABA Imbalance Following Traumatic Brain Injury. Curr. Neurol. Neurosci. Rep..

[166] Ramalingayya G., Nayak P., Shenoy R., Mallik S., Gourishetti K., Hussain S., Rao C., Nandakumar K. (2018). Naringin Ameliorates Doxorubicin-Induced Neurotoxicity In Vitro and Cognitive Dysfunction In Vivo. Phcog. Mag..

[167] Chen T., Dai Y., Hu C., Lin Z., Wang S., Yang J., Zeng L., Li S., Li W. (2024). Cellular and Molecular Mechanisms of the Blood-Brain Barrier Dysfunction in Neurodegenerative Diseases. Fluids Barriers CNS.

[168] Bu S., Nguyen H.C., Nikfarjam S., Michels D.C.R., Rasheed B., Maheshkumar S., Singh S., Singh K.K. (2022). Endothelial Cell-Specific Loss of eNOS Differentially Affects Endothelial Function. PLoS ONE.

[169] Knox E.G., Aburto M.R., Clarke G., Cryan J.F., O’Driscoll C.M. (2022). The Blood-Brain Barrier in Aging and Neurodegeneration. Mol. Psychiatry.

[170] Hadi H.A.R., Carr C.S., Al Suwaidi J. (2005). Endothelial Dysfunction: Cardiovascular Risk Factors, Therapy, and Outcome. Vasc. Health Risk Manag..

[171] Sun H.-J., Wu Z.-Y., Nie X.-W., Bian J.-S. (2019). Role of Endothelial Dysfunction in Cardiovascular Diseases: The Link Between Inflammation and Hydrogen Sulfide. Front. Pharmacol..

[172] He H., Wang L., Qiao Y., Zhou Q., Li H., Chen S., Yin D., Huang Q., He M. (2019). Doxorubicin Induces Endotheliotoxicity and Mitochondrial Dysfunction via ROS/eNOS/NO Pathway. Front. Pharmacol..

[173] Mu H., Liu H., Zhang J., Huang J., Zhu C., Lu Y., Shi Y., Wang Y. (2019). Ursolic Acid Prevents Doxorubicin-Induced Cardiac Toxicity in Mice through eNOS Activation and Inhibition of eNOS Uncoupling. J. Cell. Mol. Med..

[174] Hopfner K.-P., Hornung V. (2020). Molecular Mechanisms and Cellular Functions of cGAS-STING Signalling. Nat. Rev. Mol. Cell Biol..

[175] Wang S., Kotamraju S., Konorev E., Kalivendi S., Joseph J., Kalyanaraman B. (2002). Activation of Nuclear Factor-kappaB during Doxorubicin-Induced Apoptosis in Endothelial Cells and Myocytes Is pro-Apoptotic: The Role of Hydrogen Peroxide. Biochem. J..

[176] Graziani S., Scorrano L., Pontarin G. (2022). Transient Exposure of Endothelial Cells to Doxorubicin Leads to Long-Lasting Vascular Endothelial Growth Factor Receptor 2 Downregulation. Cells.

[177] Luu A.Z., Luu V.Z., Chowdhury B., Kosmopoulos A., Pan Y., Al-Omran M., Quan A., Teoh H., Hess D.A., Verma S. (2021). Loss of Endothelial Cell-Specific Autophagy-Related Protein 7 Exacerbates Doxorubicin-Induced Cardiotoxicity. Biochem. Biophys. Rep..

[178] Dhulkifle H., Therachiyil L., Hasan M.H., Sayed T.S., Younis S.M., Korashy H.M., Yalcin H.C., Maayah Z.H. (2024). Inhibition of Cytochrome P450 Epoxygenase Promotes Endothelium-to-Mesenchymal Transition and Exacerbates Doxorubicin-Induced Cardiovascular Toxicity. Mol. Biol. Rep..

[179] Deng C.-X., Wang R.-H. (2003). Roles of BRCA1 in DNA Damage Repair: A Link between Development and Cancer. Hum. Mol. Genet..

[180] O’Donovan P.J., Livingston D.M. (2010). BRCA1 and BRCA2: Breast/Ovarian Cancer Susceptibility Gene Products and Participants in DNA Double-Strand Break Repair. Carcinogenesis.

[181] Baretta Z., Mocellin S., Goldin E., Olopade O.I., Huo D. (2016). Effect of BRCA Germline Mutations on Breast Cancer Prognosis: A Systematic Review and Meta-Analysis. Medicine.

[182] Nyberg T., Frost D., Barrowdale D., Evans D.G., Bancroft E., Adlard J., Ahmed M., Barwell J., Brady A.F., Brewer C. (2020). Prostate Cancer Risks for Male BRCA1 and BRCA2 Mutation Carriers: A Prospective Cohort Study. Eur. Urol..

[183] Ibrahim M., Yadav S., Ogunleye F., Zakalik D. (2018). Male BRCA Mutation Carriers: Clinical Characteristics and Cancer Spectrum. BMC Cancer.

[184] Hagemeister F.B., Buzdar A.U., Luna M.A., Blumenschein G.R. (1980). Causes of Death in Breast Cancer: A Clinicopathologic Study. Cancer.

[185] Madabhushi R., Pan L., Tsai L.-H. (2014). DNA Damage and Its Links to Neurodegeneration. Neuron.

[186] Nouspikel T. (2007). DNA Repair in Differentiated Cells: Some New Answers to Old Questions. Neuroscience.

[187] Fortini P., Dogliotti E. (2010). Mechanisms of Dealing with DNA Damage in Terminally Differentiated Cells. Mutat. Res..

[188] Suberbielle E., Djukic B., Evans M., Kim D.H., Taneja P., Wang X., Finucane M., Knox J., Ho K., Devidze N. (2015). DNA Repair Factor BRCA1 Depletion Occurs in Alzheimer Brains and Impairs Cognitive Function in Mice. Nat. Commun..

[189] Li D., Bi F.-F., Chen N.-N., Cao J.-M., Sun W.-P., Zhou Y.-M., Li C.-Y., Yang Q. (2014). A Novel Crosstalk between BRCA1 and Sirtuin 1 in Ovarian Cancer. Sci. Rep..

[190] Kaneko M., Imaizumi K., Saito A., Kanemoto S., Asada R., Matsuhisa K., Ohtake Y. (2017). ER Stress and Disease: Toward Prevention and Treatment. Biol. Pharm. Bull..

[191] Wezyk M., Zekanowski C. (2018). Role of BRCA1 in Neuronal Death in Alzheimer’s Disease. ACS Chem. Neurosci..

[192] Wezyk M., Szybinska A., Wojsiat J., Szczerba M., Day K., Ronnholm H., Kele M., Berdynski M., Peplonska B., Fichna J.P. (2018). Overactive BRCA1 Affects Presenilin 1 in Induced Pluripotent Stem Cell-Derived Neurons in Alzheimer’s Disease. J. Alzheimer’s Dis..

[193] Mano T., Nagata K., Nonaka T., Tarutani A., Imamura T., Hashimoto T., Bannai T., Koshi-Mano K., Tsuchida T., Ohtomo R. (2017). Neuron-Specific Methylome Analysis Reveals Epigenetic Regulation and Tau-Related Dysfunction of BRCA1 in Alzheimer’s Disease. Proc. Natl. Acad. Sci. USA.

[194] Anderson A.J., Stoltzner S., Lai F., Su J., Nixon R.A. (2000). Morphological and Biochemical Assessment of DNA Damage and Apoptosis in Down Syndrome and Alzheimer Disease, and Effect of Postmortem Tissue Archival on TUNEL. Neurobiol. Aging.

[195] Su J.H., Satou T., Anderson A.J., Cotman C.W. (1996). Up-Regulation of Bcl-2 Is Associated with Neuronal DNA Damage in Alzheimer’s Disease. Neuroreport.

[196] Nakanishi A., Minami A., Kitagishi Y., Ogura Y., Matsuda S. (2015). BRCA1 and P53 Tumor Suppressor Molecules in Alzheimer’s Disease. Int. J. Mol. Sci..

[197] Schapira A.H., Jenner P. (2011). Etiology and Pathogenesis of Parkinson’s Disease. Mov. Disord..

[198] Gandhi S., Wood N.W. (2005). Molecular Pathogenesis of Parkinson’s Disease. Hum. Mol. Genet..

[199] Illuzzi J.L., Vickers C.A., Kmiec E.B. (2011). Modifications of P53 and the DNA Damage Response in Cells Expressing Mutant Form of the Protein Huntingtin. J. Mol. Neurosci..

[200] de la Monte S.M., Sohn Y.K., Ganju N., Wands J.R. (1998). P53- and CD95-Associated Apoptosis in Neurodegenerative Diseases. Lab. Investig..

[201] Sawa A. (2001). Alteration of Gene Expression in Down’s Syndrome (DS) Brains: Its Significance in Neurodegeneration. Protein Expression in Down Syndrome Brain.

[202] Martin L.J. (2000). P53 Is Abnormally Elevated and Active in the CNS of Patients with Amyotrophic Lateral Sclerosis. Neurobiol. Dis..

[203] Eve D.J., Dennis J.S., Citron B.A. (2007). Transcription Factor P53 in Degenerating Spinal Cords. Brain Res..

[204] Herold S., Kumar P., Wichert S.P., Kretzschmar B., Bähr M., Rossner M.J., Hein K. (2015). Neurodegeneration in Autoimmune Optic Neuritis Is Associated with Altered APP Cleavage in Neurons and Up-Regulation of P53. PLoS ONE.

[205] Rossi S., Motta C., Studer V., Macchiarulo G., Volpe E., Barbieri F., Ruocco G., Buttari F., Finardi A., Mancino R. (2014). Interleukin-1β Causes Excitotoxic Neurodegeneration and Multiple Sclerosis Disease Progression by Activating the Apoptotic Protein P53. Mol. Neurodegener..

[206] Bowles E.J.A., Walker R.L., Anderson M.L., Dublin S., Crane P.K., Larson E.B. (2017). Risk of Alzheimer’s Disease or Dementia Following a Cancer Diagnosis. PLoS ONE.

[207] Nudelman K.N.H., Risacher S.L., West J.D., McDonald B.C., Gao S., Saykin A.J. (2014). Alzheimer’s Disease Neuroimaging Initiative Association of Cancer History with Alzheimer’s Disease Onset and Structural Brain Changes. Front. Physiol..

[208] Kesler S.R., Rao V., Ray W.J., Rao A. (2017). Alzheimer’s Disease Neuroimaging Initiative Probability of Alzheimer’s Disease in Breast Cancer Survivors Based on Gray-Matter Structural Network Efficiency. Alzheimer’s Dement..

[209] Klus P., Cirillo D., Botta Orfila T., Gaetano Tartaglia G. (2015). Neurodegeneration and Cancer: Where the Disorder Prevails. Sci. Rep..

[210] Hima Bindu A., Aliya S., Jeevani T. (2011). Genetic and Degenerative Neurological Disorders ? An Emphasis on Alzheimer’s, the Mystery. J. Genet. Syndr. Gene Ther..

[211] Du L., Pertsemlidis A. (2011). Cancer and Neurodegenerative Disorders: Pathogenic Convergence through microRNA Regulation. J. Mol. Cell Biol..

[212] Ariga H. (2015). Common Mechanisms of Onset of Cancer and Neurodegenerative Diseases. Biol. Pharm. Bull.

[213] Hopkinson J.B., Milton R., King A., Edwards D. (2016). People with Dementia: What Is Known about Their Experience of Cancer Treatment and Cancer Treatment Outcomes? A Systematic Review. Psychooncology.

[214] Huang H.-K., Hsieh J.-G., Hsieh C.-J., Wang Y.-W. (2017). Do Cancer Patients with Dementia Receive Less Aggressive Treatment in End-of-Life Care? A Nationwide Population-Based Cohort Study. Oncotarget.

[215] Potapova A., Hoffman A.M., Godwin A.K., Al-Saleem T., Cairns P. (2008). Promoter Hypermethylation of the PALB2 Susceptibility Gene in Inherited and Sporadic Breast and Ovarian Cancer. Cancer Res..

[216] Thorstenson Y.R., Roxas A., Kroiss R., Jenkins M.A., Yu K.M., Bachrich T., Muhr D., Wayne T.L., Chu G., Davis R.W. (2003). Contributions of ATM Mutations to Familial Breast and Ovarian Cancer. Cancer Res..

[217] Renwick A., Thompson D., Seal S., Kelly P., Chagtai T., Ahmed M., North B., Jayatilake H., Barfoot R., Spanova K. (2006). ATM Mutations That Cause Ataxia-Telangiectasia Are Breast Cancer Susceptibility Alleles. Nat. Genet..

[218] Yang X., Song H., Leslie G., Engel C., Hahnen E., Auber B., Horváth J., Kast K., Niederacher D., Turnbull C. (2020). Ovarian and Breast Cancer Risks Associated With Pathogenic Variants in RAD51C and RAD51D. J. Natl. Cancer Inst..

[219] Moyer C.L., Ivanovich J., Gillespie J.L., Doberstein R., Radke M.R., Richardson M.E., Kaufmann S.H., Swisher E.M., Goodfellow P.J. (2020). Rare BRIP1 Missense Alleles Confer Risk for Ovarian and Breast Cancer. Cancer Res..

[220] Shimelis H., LaDuca H., Hu C., Hart S.N., Na J., Thomas A., Akinhanmi M., Moore R.M., Brauch H., Cox A. (2018). Triple-Negative Breast Cancer Risk Genes Identified by Multigene Hereditary Cancer Panel Testing. J. Natl. Cancer Inst..

[221] Boonen R.A.C.M., Wiegant W.W., Celosse N., Vroling B., Heijl S., Kote-Jarai Z., Mijuskovic M., Cristea S., Solleveld-Westerink N., van Wezel T. (2022). Functional Analysis Identifies Damaging CHEK2 Missense Variants Associated with Increased Cancer Risk. Cancer Res..

[222] Prakash R., Zhang Y., Feng W., Jasin M. (2015). Homologous Recombination and Human Health: The Roles of BRCA1, BRCA2, and Associated Proteins. Cold Spring Harb. Perspect. Biol..

[223] Plun-Favreau H., Lewis P.A., Hardy J., Martins L.M., Wood N.W. (2010). Cancer and Neurodegeneration: Between the Devil and the Deep Blue Sea. PLoS Genet..

[224] Hoch N.C., Hanzlikova H., Rulten S.L., Tétreault M., Komulainen E., Ju L., Hornyak P., Zeng Z., Gittens W., Rey S.A. (2017). XRCC1 Mutation Is Associated with PARP1 Hyperactivation and Cerebellar Ataxia. Nature.

[225] Wang C.-Y., Deneen B., Tzeng S.-F. (2019). BRCA1/BRCA2-Containing Complex Subunit 3 Controls Oligodendrocyte Differentiation by Dynamically Regulating Lysine 63-Linked Ubiquitination. Glia.

[226] Szybińska A., Leśniak W. (2017). P53 Dysfunction in Neurodegenerative Diseases—The Cause or Effect of Pathological Changes?. Aging Dis..

[227] Chang J.R., Ghafouri M., Mukerjee R., Bagashev A., Chabrashvili T., Sawaya B.E. (2012). Role of P53 in Neurodegenerative Diseases. Neurodegener Dis..

[228] Saha S., Mandal P., Ganguly S., Jana D., Ayaz A., Banerjee A., Chouhan R., Sarkar D.K. (2015). Decreased Expression of BRCA2 Accelerates Sporadic Breast Cancer Progression. Indian J. Surg. Oncol..

[229] Loredo-Pozos G., Chiquete E., Oceguera-Villanueva A., Panduro A., Siller-López F., Ramos-Márquez M.E. (2009). Expression Profile of BRCA1 and BRCA2 Genes in Premenopausal Mexican Women with Breast Cancer: Clinical and Immunohistochemical Correlates. Med. Oncol..

[230] Melchor L., Benítez J. (2013). The Complex Genetic Landscape of Familial Breast Cancer. Hum. Genet..

[231] Yoshida R. (2021). Hereditary Breast and Ovarian Cancer (HBOC): Review of Its Molecular Characteristics, Screening, Treatment, and Prognosis. Breast Cancer.

[232] Mai P.L., Chatterjee N., Hartge P., Tucker M., Brody L., Struewing J.P., Wacholder S. (2009). Potential Excess Mortality in BRCA1/2 Mutation Carriers beyond Breast, Ovarian, Prostate, and Pancreatic Cancers, and Melanoma. PLoS ONE.

[233] Guo M., Wang S.M. (2022). The BRCAness Landscape of Cancer. Cells.

[234] Liu L., Zhou W., Cheng C.-T., Ren X., Somlo G., Fong M.Y., Chin A.R., Li H., Yu Y., Xu Y. (2014). TGFβ Induces “BRCAness” and Sensitivity to PARP Inhibition in Breast Cancer by Regulating DNA-Repair Genes. Mol. Cancer Res..

[235] Singh S., Nguyen H., Michels D., Bazinet H., Matkar P.N., Liu Z., Esene L., Adam M., Bugyei-Twum A., Mebrahtu E. (2020). BReast CAncer Susceptibility Gene 2 Deficiency Exacerbates Oxidized LDL-induced DNA Damage and Endothelial Apoptosis. Physiol. Rep..

[236] Komirishetty P., Areti A., Yerra V.G., Ruby P.K., Sharma S.S., Gogoi R., Sistla R., Kumar A. (2016). PARP Inhibition Attenuates Neuroinflammation and Oxidative Stress in Chronic Constriction Injury Induced Peripheral Neuropathy. Life Sci..

[237] Nasr M.M., Wahdan S.A., El-Naga R.N., Salama R.M. (2024). Neuroprotective Effect of Empagliflozin against Doxorubicin-Induced Chemobrain in Rats: Interplay between SIRT-1/MuRF-1/PARP-1/NLRP3 Signaling Pathways and Enhanced Expression of miRNA-34a and LncRNA HOTAIR. Neurotoxicology.

